# Global Burden of Aortic Aneurysm and Attributable Risk Factors from 1990 to 2017

**DOI:** 10.5334/gh.920

**Published:** 2021-05-04

**Authors:** Linyan Wei, Xiang Bu, Xiqiang Wang, Jing Liu, Aiqun Ma, Tingzhong Wang

**Affiliations:** 1 Department of Cardiovascular Medicine, the First Affiliated Hospital of Xi’an Jiaotong University, Xi’an 710061, Shaanxi, CN; 2 Department of Respiratory and Critical Care Medicine, the First Affiliated Hospital of Xi’an Jiaotong University, Xi’an 710061, Shaanxi, CN; 3 Key Laboratory of Molecular Cardiology, Shaanxi Province, CN; 4 Key Laboratory of Environment and Genes Related to Diseases (Xi’an Jiaotong University), Ministry of Education, CN

**Keywords:** aortic aneurysm, global burden of disease, deaths, disability-adjusted life years, risk factors

## Abstract

**Background:**

To date, our understanding of the global aortic aneurysm (AA) burden distribution is very limited.

**Objective:**

To assess a full view of global AA burden distribution and attributable risk factors from 1990 to 2017.

**Methods:**

We extracted data of AA deaths, disability-adjusted life years (DALYs), and their corresponding age-standardized rates (ASRs), in general and by age/sex from the 2017 Global Burden of Disease (GBD) study. The current AA burden distribution in 2017 and its changing trend from 1990 to 2017 were separately showed. The spatial divergence was discussed from four levels: global, five social-demographic index regions, 21 GBD regions, and 195 countries and territories. We also estimated the risk factors attributable to AA related deaths.

**Results:**

Globally, the AA deaths were 167,249 with an age-standardized death rate (ASDR) of 2.19/100,000 persons in 2017, among which the elderly and the males accounted for the majority. Although reductions in ASRs were observed in developed areas, AA remained an important health issue in those relatively underdeveloped areas and might be much more important in the near future. AA may increasingly affect the elderly and the female population. Similar patterns of AA DALYs burden were noted during the study period. AA burden attributable to high blood pressure and smoking decreased globally and there were many heterogeneities in their distribution.

**Discussion:**

AA maintained an incremental public health issue worldwide. The change pattern of AA burden was heterogeneous across locations, ages, and sexes and it is paramount to improve resource allocation for more effective and targeted prevention strategies. Also, prevention of tobacco consumption and blood pressure control should be emphasized.

## Introduction

An aortic aneurysm (AA) is a focal dilatation of the aorta to greater than 1.5 times normal size. The major complications of AA are catastrophic dissections and ruptures, which are usually surgical emergencies and have an ensuing mortality of 90% [1] even with prompt treatment. AA-related mortalities are estimated at about 200,000 per year worldwide [2], representing a considerable public health concern.

Thus far, most of our knowledge of AA burden distribution is largely hampered to epidemiological surveys executed in individual developed countries, for an outdated period, and limited to a defined age group/gender. For example, it’s reported that abdominal AA caused 1.3% of all deaths among men aged 65–85 years in developed countries [3]. Therefore, we currently only get sporadic information on the entire blueprint of AA global burden.

The Global Burden of Disease (GBD) study is an ongoing global collaboration that uses all available epidemiological data to provide a rigorous and comparable measurement of the burden of 328 diseases across 195 countries and territories [4,5,6]. The Guidelines for Accurate and Transparent Health Estimates Reporting promote best practices in reporting health estimates [7]. It provided an opportunity to comprehensively assess the distribution and development trends of AA burden and then enable policymakers to make informed decisions about how to allocate resources to best improve population health. In a previous study, Sampson et al. described the mortality trends of aortic dissection and aneurysms using the data derived from the GBD Study 2010 [8]. As of now, no more updated research has been reported. Moreover, it is unclear whether AA has undergone epidemiological transitions so far, with the remarkable aging of the population and the changing burden of the related risk factors.

To bridge those knowledge gaps and complete the torn puzzle, we conducted this study to systematically reveal the levels and trends of global mortality, disability-adjusted life years (DALY) of AA, and the major risk factors, according to locations, ages, and sexes, based on updated data from the GBD study from 1990 to 2017, which may potentially inform health policy decisions.

## Methods

### Study data

Information on annual AA deaths, DALYs, and respective age-standardized rate (ASR), by locations, ages, and sexes, from 1990 to 2017, were retrieved from the Global Health Data Exchange (GHDx) query tool (http://ghdx.healthdata.org/gbd-results-tool) [9]. The general methods for the GBD study and for the estimation of AA burden have been detailed in previous studies [8,10].

To analyze the global AA burden distribution, we classified the location information into three levels. Firstly, we used the social-demographic-index (SDI) to categorize the countries and territories into five SDI quintiles (high, high-middle, middle, low-middle, and low). Secondly, as shown in Tables 1 and 2, the world was geographically divided into 21 GBD regions. Lastly, we showed AA burden in 195 countries and territories by drawing world maps.

**Table 1:** The death cases and age-standardized death rate of aortic aneurysm in 1990 and 2017, and its temporal trends from 1990 to 2017.

Characteristics	1990		2017		1990–2017

	Death cases No. × 10^3^ (95% UI)	ASR per 100,000 No. (95% UI)	Death cases No. × 10^3^ (95% UI)	ASR per 100,000 No. (95% UI)	EAPC No. (95% UI)

**Overall**	104.8 (101.1–110.6)	2.88 (2.79–3.03)	167.2 (159.8–174.1)	2.19 (2.09–2.28)	–1.32 (–1.43 to –1.21)
**Sex**					
**Male**	69.4 (65.6–75.8)	4.43 (4.21–4.79)	106.3 (100.0–113.8)	3.15 (2.97–3.35)	–1.59 (–1.71 to –1.48)
**Female**	35.4 (33.6–39.1)	1.75 (1.67–1.92)	60.9 (58.7–65.3)	1.41 (1.36–1.52)	–1.06 (–1.16 to –0.96)
**Social-demographic index**					
**Low**	5.7 (4.4–7.9)	2.08 (1.61–2.79)	10.9 (9.2–13.4)	1.82 (1.53–2.23)	–0.71 (–0.90 to –0.52)
**Low–middle**	8.6 (7.4–10.5)	1.81 (1.56–2.19)	19.0 (17.2–21.3)	1.85 (1.66–2.07)	–0.09 (–0.19 to 0.00)
**Middle**	12.6 (11.5–14.2)	1.57 (1.42–1.75)	29.8 (26.9–32.2)	1.50 (1.36–1.62)	–0.48 (–0.59 to –0.37)
**High–middle**	18.5 (17.7–19.6)	2.12 (2.03–2.25)	34.5 (33.1–36.2)	2.01 (1.93–2.11)	–0.43 (–0.55 to –0.31)
**High**	59.2 (58.4–60.5)	4.47 (4.41–4.56)	72.7 (70.8–74.4)	2.94 (2.86–3.01)	–1.92 (–2.08 to –1.76)
**Region**					
**Central Asia**	0.6 (0.6–0.7)	1.40 (1.24–1.60)	1.4 (1.4–1.5)	2.19 (2.10–2.28)	1.67 (1.52 to 1.82)
**Central Europe**	3.6 (3.4–3.9)	2.47 (2.31–2.66)	5.8 (5.5–6.0)	2.67 (2.56–2.78)	0.43 (0.27 to 0.59)
**Eastern Europe**	6.9 (6.5–7.3)	2.46 (2.33–2.61)	10.9 (10.5–11.2)	3.15 (3.05–3.26)	0.64 (0.40 to 0.87)
**Australasia**	1.9 (1.8–1.9)	7.58 (7.34–7.82)	1.7 (1.6–1.8)	3.18 (2.91–3.45)	–3.86 (–4.09 to –3.64)
**High–income Asia Pacific**	6.5 (6.4–6.7)	3.47 (3.40–3.56)	20.6 (19.5–21.4)	3.80 (3.61–3.97)	0.67 (0.50 to 0.83)
**High–income North America**	15.0 (14.5–15.5)	4.88 (4.81–4.95)	18.5 (18.3–18.8)	2.35 (2.28–2.43)	–3.44 (–3.74 to –3.15)
**Southern Latin America**	2.7 (2.5–3.0)	5.92 (5.46–6.54)	3.5 (3.2–3.8)	4.15 (3.82–4.53)	–1.60 (–1.79 to –1.41)
**Western Europe**	29.8 (29.2–30.6)	4.80 (4.71–4.92)	30.9 (29.9–32.0)	2.99 (2.89–3.09)	–2.25 (–2.51 to –1.99)
**Andean Latin America**	0.3 (0.3–0.4)	1.70 (1.42–2.04)	0.8 (0.7–0.9)	1.48 (1.34–1.63)	–0.58 (–0.68 to –0.48)
**Caribbean**	1.0 (0.9–1.1)	3.80 (3.46–4.21)	1.5 (1.4–1.6)	2.97 (2.74–3.25)	–1.18 (–1.29 to –1.07)
**Central Latin America**	1.6 (1.6–1.7)	2.04 (1.99–2.12)	3.5 (3.3–3.7)	1.55 (1.47–1.64)	–1.53 (–1.72 to –1.35)
**Tropical Latin America**	3.5 (3.5–3.6)	4.06 (3.97–4.15)	10.1 (9.6–10.5)	4.46 (4.26–4.63)	0.12 (–0.02 to 0.26)
**North Africa and Middle East**	2.4 (2.0–3.5)	1.57 (1.30–2.27)	5.7 (5.2–7.1)	1.51 (1.38–1.91)	–0.15 (–0.28 to –0.02)
**South Asia**	8.0 (6.2–10.9)	1.67 (1.34–2.26)	21.7 (18.0–25.8)	1.91 (1.58–2.26)	0.39 (0.24 to 0.53)
**East Asia**	8.0 (6.5–9.7)	0.99 (0.80–1.18)	17.4 (15.8–19.2)	0.92 (0.83–1.01)	–0.53 (–0.67 to –0.39)
**Oceania**	0.1 (0.1–0.2)	4.93 (4.01–6.30)	0.2 (0.2–0.3)	4.26 (3.50–5.34)	–0.79 (–0.90 to –0.68)
**Southeast Asia**	3.7 (3.3–4.3)	1.79 (1.59–2.08)	9.0 (8.1–10.2)	1.83 (1.64–2.06)	–0.19 (–0.31 to –0.07)
**Central Sub–Saharan Africa**	3.7 (3.3–4.3)	4.17 (2.86–5.79)	9.0 (8.1–10.2)	2.85 (2.21–3.58)	–1.79 (–2.02 to –1.57)
**Eastern Sub–Saharan Africa**	2.2 (1.5–3.0)	3.37 (2.24–4.55)	2.8 (2.0–3.5)	2.09 (1.52–2.67)	–2.30 (–2.54 to –2.06)
**Southern Sub–Saharan Africa**	0.8 (0.6–0.9)	3.08 (2.40–3.58)	1.2 (1.1–1.4)	2.44 (2.27–2.84)	–1.24 (–1.87 to –0.61)
**Western Sub–Saharan Africa**	1.9 (1.5–2.3)	2.43 (1.91–2.96)	2.5 (2.1–3.1)	1.64 (1.35–2.02)	–1.88 (–2.11 to –1.66)

ASR: Age-standardized rate; EAPC: Estimated annual percentage change.

**Table 2:** The DALYs and age-standardized DALYs rate of aneurysm in 1990 and 2017, and its temporal trends from 1990 to 2017.

Characteristics	1990		2017		1990–2017

	DALYs No. × 10^3^ (95% UI)	ASR per 100,000 No. (95% UI)	DALYs No. × 10^3^ (95% UI)	ASR per 100,000 No. (95% UI)	EAPC No. (95% UI)

**Overall**	2089.42 (1996.56–2240.68)	51.09 (49.01–54.52)	3039.86 (2877.17–3186.41)	38.18 (36.21–40)	–1.40 (–1.51 to –1.29)
**Sex**					
**Male**	1457.54 (1370.65–1621.92)	78.08 (73.62–86.10)	2091.23 (1946.02–2249.36)	56.00 (52.27–60.17)	–1.56 (–1.67 to –1.44)
**Female**	631.88 (582.34–731.90)	28.83 (26.73–33.15)	948.62 (907.06–1049.58)	22.34 (21.36–24.74)	–1.26 (–1.37 to –1.14)
**Social-demographic index**					
**Low**	142.67 (103.11–200.88)	41.06 (31–56.71)	250.79 (209.43–313.40)	34.52 (29–42.72)	–0.89 (–1.09 to –0.70)
**Low-middle**	203.55 (169.67–251.85)	34.57 (29.41–42.6)	421.06 (378.61–475.98)	34.89 (31.54–39.3)	–0.16 (–0.26 to –0.06)
**Middle**	299.33 (274.10–345.30)	29.69 (27.07–33.75)	608.42 (548.59–660.43)	27.72 (25.02–30.06)	–0.59 (–0.72 to –0.46)
**High-middle**	430.05 (412.08–459.38)	44.17 (42.35–47.05)	709.83 (677.80–742.50)	39.64 (37.83–41.49)	–0.67 (–0.81 to –0.53)
**High**	1008.90 (993.83–1030.02)	77.74 (76.57–79.32)	1041.69 (1012.52–1068.82)	49.11 (47.71–50.43)	–2.07 (–2.22 to –1.91)
**Region**					
**Central Asia**	15.09 (13.88–16.85)	30.11 (27.5–33.86)	31.78 (30.37–33.39)	42.18 (40.42–44.09)	1.19 (1.00 to 1.39)
**Central Europe**	82.98 (77.44–89.70)	55.31 (51.6–59.78)	111.01 (105.78–115.58)	56.05 (53.43–58.4)	0.15 (–0.04 to 0.33)
**Eastern Europe**	161.14 (151.09–172.25)	56.67 (53.1–60.51)	234.70 (226.45–242.93)	71.73 (69.13–74.43)	0.58 (0.31 to 0.85)
**Australasia**	30.90 (29.85–31.97)	125.05 (121.01–129.05)	23.20 (21.21–25.33)	48.06 (43.89–52.78)	–4.18 (–4.41 to –3.96)
**High-income Asia Pacific**	111.04 (108.60–114.17)	54.89 (53.68–56.46)	251.43 (237.58–263.76)	57.55 (54.23–60.79)	0.49 (0.33 to 0.66)
**High-income North America**	320.64 (315.67–325.75)	88.25 (86.85–89.7)	249.77 (240.96–258.48)	43.99 (42.33–45.64)	–3.29 (–3.57 to –3.02)
**Southern Latin America**	51.75 (47.66–57.30)	109.14 (100.63–120.8)	59.53 (54.46–65.19)	73.37 (67.06–80.34)	–1.77 (–1.95 to –1.59)
**Western Europe**	488.44 (479.16–500.08)	82.44 (80.88–84.15)	427.70 (411.78–443.29)	48.96 (46.95–50.87)	–2.43 (–2.67 to –2.18)
**Andean Latin America**	7.72 (6.38–9.26)	33.7 (27.84–40.58)	15.24 (13.64–16.93)	27.77 (24.82–30.81)	–0.74 (–0.85 to –0.63)
**Caribbean**	17.89 (16.17–19.89)	66.57 (60.19–74.11)	26.93 (24.68–29.51)	53.19 (48.75–58.26)	–1.09 (–1.19 to –0.98)
**Central Latin America**	35.90 (34.89–37.29)	39.09 (37.97–40.66)	66.74 (62.76–70.82)	28.56 (26.87–30.29)	–1.69 (–1.87 to –1.51)
**Tropical Latin America**	89.79 (87.68–92.08)	87.73 (85.73–89.92)	213.43 (203.49–222.23)	90.65 (86.45–94.36)	–0.17 (–0.33 to 0.00)
**North Africa and Middle East**	58.81 (46.17–85.06)	31.54 (25.39–45.71)	127.84 (116.62–159.71)	29.17 (26.58–36.71)	–0.30 (–0.41 to –0.19)
**South Asia**	189.69 (141.74–261.26)	31.26 (24.11–42.87)	466.14 (388.16–555.97)	34.47 (28.6–41.02)	0.24 (0.08 to 0.40)
**East Asia**	203.69 (169.41–258.66)	20.59 (16.96–25.63)	364.35 (328.39–401.38)	18.06 (16.27–19.84)	–0.81 (–1.01 to –0.60)
**Oceania**	3.59 (2.47–4.96)	106.02 (78.35–143.28)	6.99 (4.95–9.52)	91.85 (70.54–120.74)	–0.72 (–0.80 to –0.63)
**Southeast Asia**	83.79 (75.30–101.13)	31.62 (28.37–37.55)	178.72 (160.53–204.22)	31.23 (28.14–35.59)	–0.32 (–0.45 to –0.19)
**Central Sub-Saharan Africa**	19.47 (13.54–28.43)	81.53 (56.95–116.06)	29.93 (23.17–39.06)	56.49 (43.77–72.25)	–1.75 (–1.97 to –1.53)
**Eastern Sub-Saharan Africa**	55.05 (37.53–80.93)	66.76 (45.22–94.39)	69.82 (51.17–87.19)	41.08 (29.88–50.83)	–2.35 (–2.60 to –2.10)
**Southern Sub-Saharan Africa**	18.37 (15.00–20.33)	59.57 (47.49–67.47)	26.75 (24.63–30.41)	46.42 (42.85–53.12)	–1.30 (–2.04 to –0.56)
**Western Sub-Saharan Africa**	43.68 (34.13–53.97)	46.48 (36.31–56.84)	57.88 (47.83–70.51)	30.7 (25.37–37.66)	–1.97 (–2.20 to –1.75)

ASR: Age-standardized rate; DALY: Disability-adjusted life year; EAPC: Estimated annual percentage change.

### Statistical analysis

We used ASRs (age-standardized death rate [ASDR] and age-standardized DALY rate) to determine the burden of AA. ASRs (per 100,000 population) is equal to the sum of the product of the specific age ratio (*a_i_*) in age group *i* and the number (or weight) (*w_i_*) of the selected reference standard population group *i* divided by the sum of number (or weight) of the standard population, that is: ASR = \frac{{\mathop \sum \nolimits_{i = 1}^A {\rm{ }}{a_i}\;{w_i}}}{{\mathop \sum \nolimits_{i = 1}^A {\rm{ }}{w_i}}} \times 100,000. More importantly, the ASR trends can serve as a good surrogate for shifting patterns of disease within a population, and the estimated annual percentage ‘change (EAPC) is a widely used measure of the ASR trend over a specified interval. Consequently, a regression line was fitted to the natural logarithm of the rates: *y* = *α* + *βx* + ε, where *y* represents ln *ASR*, and *x* refers to the calendar year. EAPC = 100× (*exp*(*β*)–1) and its 95% uncertainty interval (UI) can also be obtained from the regression model.

Additionally, to explore the influential factors for EAPCs, we assessed the association between EAPCs and ASRs (1990)/HDI (2017) at the national level. All statistical analyses were performed using R program (Version 3.6.3, R core team). A *p* value of less than 0.05 was considered statistically significant.

## Results

### The deaths burden of AA

#### AA Deaths in 2017

**Figure 1:**
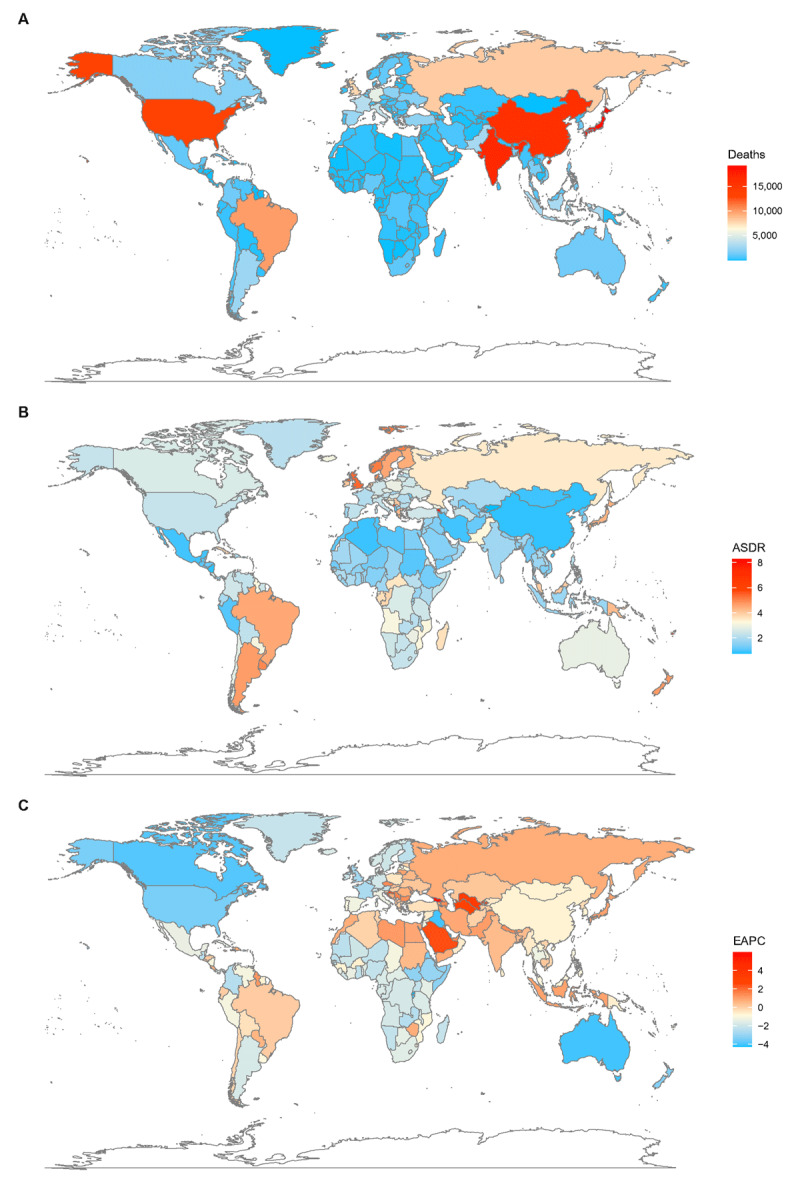
The global deaths burden of AA in 195 countries and territories. **A**. The absolute number of AA death cases in 2017. **B**. The ASDR (per 100,000 persons) of AA in 2017. **C**. The EAPC of AA ASDRs between 1990 and 2017. AA, aortic aneurysm; ASDR, age standardized death rate; EAPC, estimated annual percentage change.

Globally, the death cases of AA were 167,249 (95% UI = 174,146–159,775) with an ASDR of 2.19/100,000 persons (95% UI = 2.09–2.28) in 2017. Areas with higher SDI had more deaths; the high SDI quintile had the highest number (72,685, 95% UI = 70,796–74,353), almost half of the total number of the world. The high SDI quintile also had the highest ASDRs (2.94, 95% UI = 2.86–3.01), which was much higher than the world average. With respect to the 21 GBD regions, the highest deaths were observed in Western Europe (30,905, 95% UI = 29,882–31,987), followed by South Asia (21,694, 95% UI = 17,970–25,800). The highest ASDR of 4.46/100,000 persons (95% UI = 4.26–4.63) was observed in tropical Latin America and the lowest ASDR of 0.92/100,000 persons (95% UI = 0.83–1.01) was observed in East Asia (Table 1). Japan, India, China, and United States were the four countries with the highest reported AA death cases. Armenia, Montenegro, and Fiji showed the highest ASDRs, while Kyrgyzstan, Nicaragua, and China had the lowest (e-Table 2, Figure 1A, B).

The AA deaths varied considerably across ages and sexes, with the highest death cases observed in the 75- to 79-year-old group in males and the 80- to 84-year-old group in females. People over 50 exceeded 90% of the total deaths; people over 70 accounted for 74.26% AA deaths in females but only 59.60% in males. In all age groups under 90, more than half or even two-thirds of the death cases were recorded in males, while females over 90 have higher occurrences (Table 1, e-Figure 1A).

#### Trends of AA deaths

**Figure 2:**
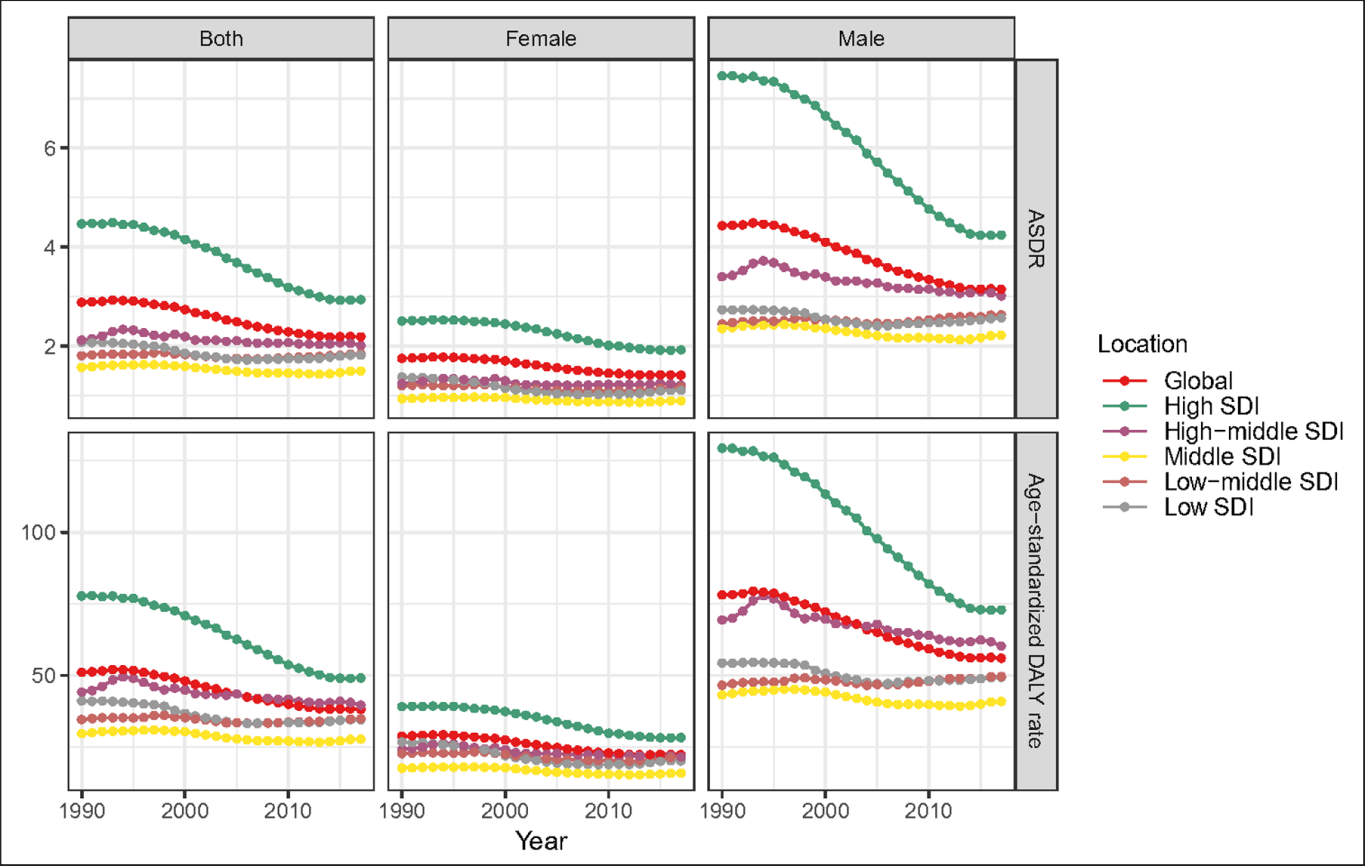
The change trends of ASDR/age-standardized DALY rate (per 100,000 persons) globally and among different SDI quintiles between 1990 and 2017. **A**. ASDR. **B**. Age standardized DALY rate. ASDR, age-standardized death rate; DALY, disability-adjusted life year.

**Figure 3:**
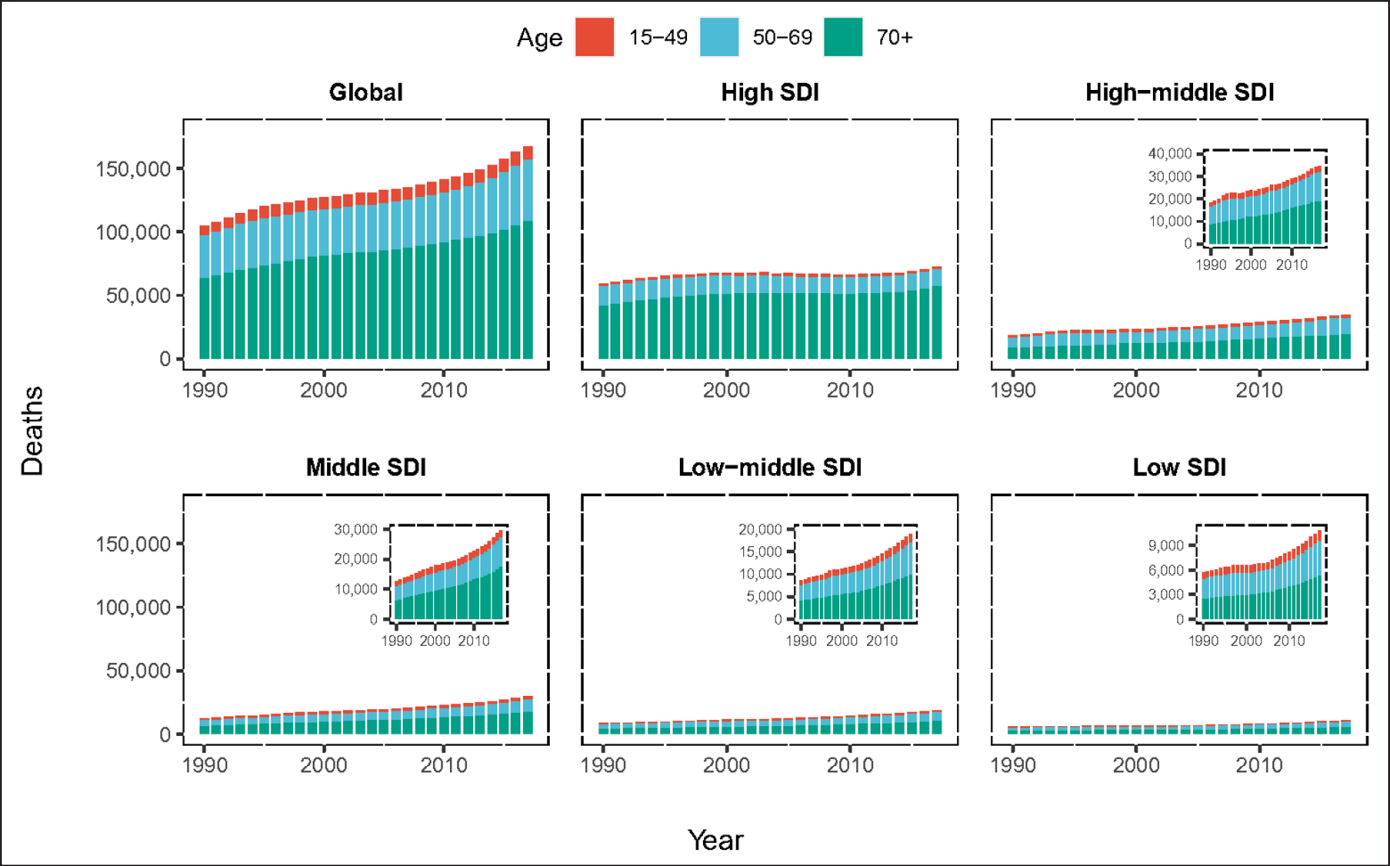
The proportion of the three age groups (15–49 years, 50–69 years and 70+ years) for aortic aneurysm deaths globally and in five SDI quintiles between 1990 and 2017. SDI: social-demographic index.

At the global level, the annual deaths increased gradually. It was noted in both sexes, but female achieved a higher increase (72.03%) than male (53.17%) (Table 1). Contrary to the 59.58% increase in overall deaths over the past 28 years, the global ASDR was decreased from 2.88/100,000 persons in 1990 to 2.19/100,000 persons in 2017, with an overall EAPC of –1.32 (95% UI = 1.43 to –1.21). The ASDR in male subjects was markedly higher and decreased more obviously than that in females (Figure 2). The proportion of the three age groups (15–49 years, 50–69 years, and 70+ years) in AA deaths remained stable between 1990 and 2017. AA-related deaths in the 70-plus age group remained the highest among the three age groups during the study period (Figure 3).

Regarding SDI level analysis, the number of AA deaths increased in all five SDI quintiles between 1990 and 2017, precipitously increased in the middle and low-middle SDI quintiles (1.35-fold and 1.21-fold, respectively) and less obviously in the high SDI quintile (0.22-fold). However, the ASDR in the high SDI quintile was on the decline with an EAPC of – 1.92 (95% UI = –2.08 to –1.76). The ASDRs in the other SDI quintiles were stable (Table 1, Figure 2).

Regionally, absolute numbers of AA deaths increased in almost all GBD regions between 1990 and 2017, except for Australasia. The most pronounced increase was observed in high-income Asia Pacific (Table 1). The AA deaths at young age (15- to 49-year-old group) remained stable when compared the data in 2017 with that in 1990. Alarmingly, the number of AA deaths in the high-income Asia Pacific region and South Asia had grown rapidly, especially among older people (over 70 years old). In other regions with a heavy burden of AA deaths, Western Europe still had a highest number of deaths, and people over 70 years old accounted for a large proportion; East Europe and East Asia had an obvious increase while the high-income North America had achieved a visible decline of deaths (e-Figure 2A, C). Only three GBD regions (Central Asia, high-income Asia Pacific, and Eastern Europe) reported increasing AA ASDRs, and six GBD regions (high-income North America, Southern Latin America, Central Latin America, Western Europe, Australasia, and Caribbean) reported decreasing AA ASDRs; the ASDRs in the other GBD regions were stable during the study period (Table 1).

On observation from the 195 countries and territories, ASDRs showed an upward trend in 45 countries and territories, a stable trend in 20 countries and territories, and a downward trend in 130 countries and territories (Figure 1C). The three countries and territories with the highest EAPC were Georgia, Uzbekistan, and Turkmenistan; the three countries and territories with the lowest EAPC were Rwanda, Qatar, and Australia. The details were listed in e-Table 2.

**Figure 4:**
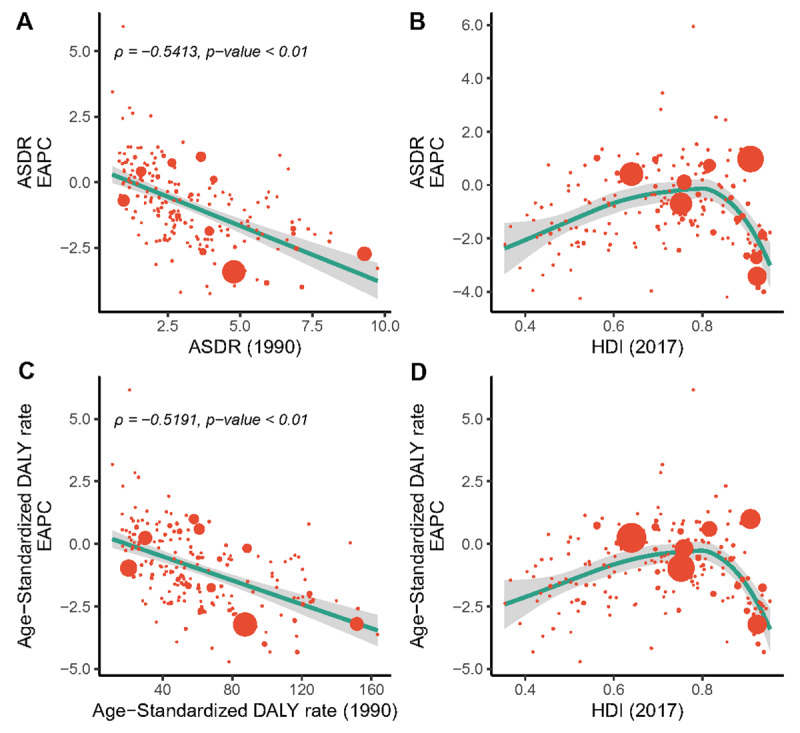
The correlation between the EAPC of deaths/DALYs and (**A, B**) the corresponding ASRs in 1990 and (**C, D**) HDI in 2017. The size of circle is increased with the number of death cases of aortic aneurysm. ASR, age-standardized death rate; DALYs, disability-adjusted life years; EAPC, estimated annual percentage change; HDI, human development index.

Our findings demonstrated a significant negative relationship between EAPC and the ASDRs in1990 (ρ = –0.5413, *p* < 0.01, Pearson correlation analysis), suggesting that those countries with lower disease reservoir at baseline have experienced a more rapid increase in ASDRs (Figure 4A). Conversely, no significant linear correlation was found between the EAPCs of ASDR and the HDI in 2017, while we can still find positive associations at the beginning and negative associations for the rest when fitting using polynomials (Figure 4B).

### The DALYs burden of AA

#### AA DALYs in 2017

Meanwhile, the global AA-related DALYs in 2017 was 3,039,858 (95% UI = 2,877,174–3,186,410), for which the high SDI quintile contributed the most. The global age-standardized DALY rate was 38.18/100,000 persons (95% UI = 36.2–40.00) in 2017. The high SDI quintile also had the highest age-standardized DALY rate, while the Middle SDI quintile had the lowest one. With respect to 21 GBD regions, the highest DALY was observed in South Asia, followed by Western Europe. The highest age-standardized DALY rate of 91.85/100,000 persons was observed in Oceania and the lowest 18.06/100,000 persons was observed in East Asia (Table 2). India, China, Japan and United States were the four countries with the highest reported AA DALYs in 2017. Armenia, Montenegro, and Fiji showed the highest age-standardized DALY rates, while Nicaragua, Kyrgyzstan, and Bahrain had the lowest ones (e-Table 3, e-Figure 3A, B). AA DALYs are similar to deaths in that they differ greatly across ages and sexes. While the peak of the DALY curves is roughly 10 years earlier than that of the deaths (e-Figure 1B).

#### Trends of AA DALYs

At the global level, a 45.49% increase was noted for DALYs over the past 28 years. In contrast, the global age-standardized DALY rate was decreased from 51.09/100,000 persons (95% UI = 49.01–54.52) in 1990 to 38.18/100,000 persons (95% UI = 36.21–40.00) in 2017, with an overall EAPC of –1.40 (95% UI = –1.51 to –1.29). A higher age-standardized DALY rate and its more pronounced decline were noted in male (Table 2).

Regarding SDI level analysis, the total DALYs increased in all SDI quintiles, with increased ranged from 1.07-fold in the low-middle SDI quintile to 0.03-fold in the high SDI quintile. Correspondingly, the age-standardized DALY rate decreased most seriously in the high SDI quintile with an EAPC of –2.07 (95% UI = –2.22 to –1.91) (Table 2, Figure 2).

Among the 21 GBD regions, Central Asia showed the largest increase of AA DALYs between 1990 and 2017. In parallel, the AA DALYs of Australasia, high-income North America and Western Europe decreased a lot. The AA DA LYs at young age (15- to 49-year-old group) remained stable when comparing the data in 2017 with that in 1990. Alarmingly, the number of AA DALYs among older people had grown sharply in most Asia areas, including South Asia, East Asia, high-income Asia Pacific, and Southeast Asia. In other regions with a large number of AA DALYs, Western Europe still had the highest number of DALYs, and people over 70 years old accounted for the largest proportion; Eastern Europe had an obvious increase while high-income North America and tropical Latin America had achieved a visible decline (e-Figure 2B, D). The age-standardized DALY rate increased in only two regions, decreased in seven regions, and remained stable in all the other regions (Table 2).

On observation from the 195 countries and territories, age-standardized DALY rates showed an upward trend in 42 countries and territories, a stable trend in 18 countries and territories, and a downward trend in 135 countries and territories (e-Figure 3C). The three countries and territories with the highest EAPC of DALYs were Georgia, Uzbekistan, and Turkmenistan; the three countries and territories with the lowest EAPC of DALYs were Rwanda, Australia, and Burundi. The details are listed in e-Table 3. The relationship between the EAPC of DALYs and age-standardized DALY rate/HDI mirrored the same pattern of the EAPC of deaths (e-Figure 4C, D).

### Attributable risk factors changes

**Figure 5:**
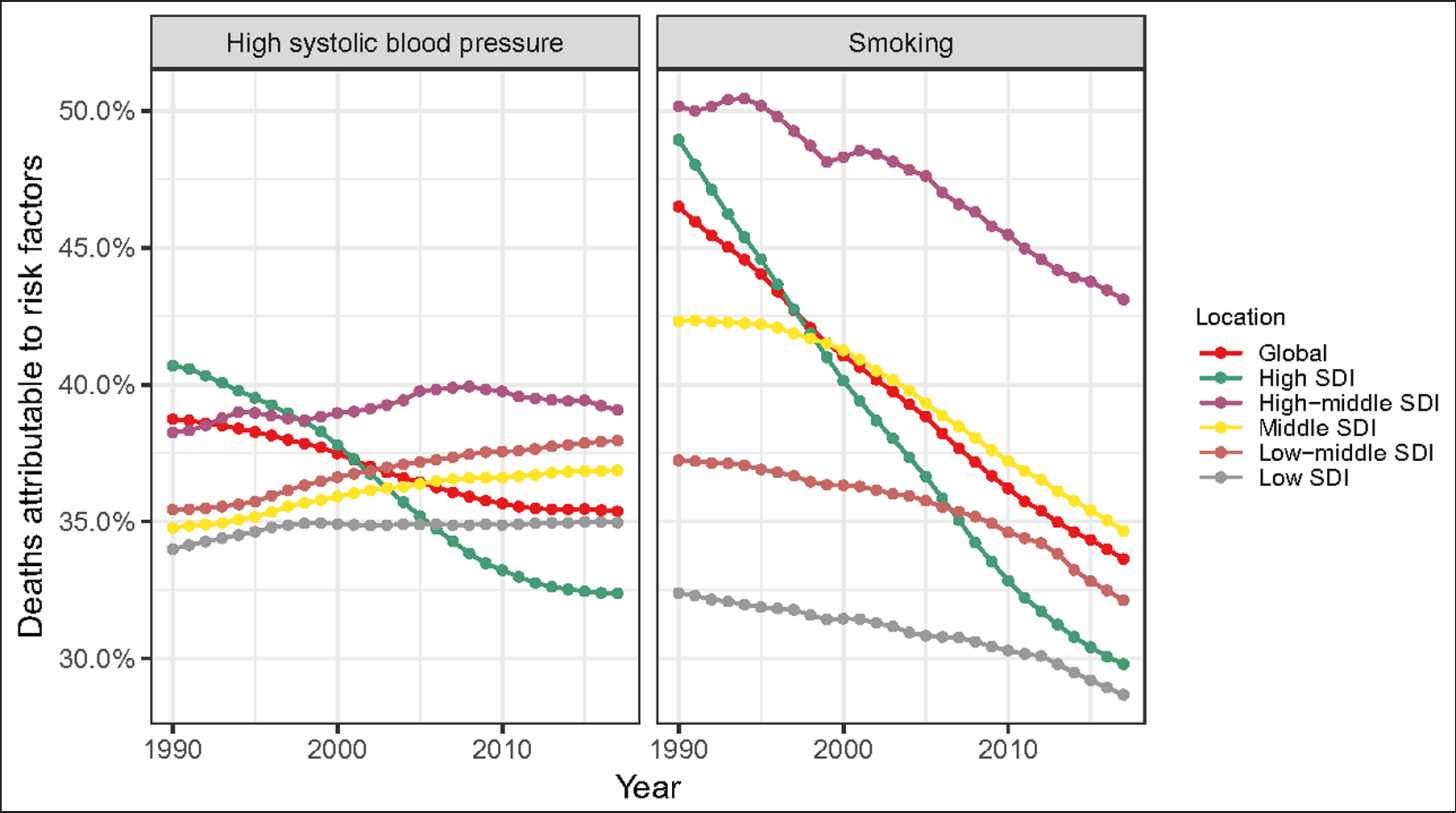
The proportion of deaths attributable to high systolic blood pressure and smoking globally and in five SDI quintiles between 1990 and 2017. SDI: social-demographic index.

High systolic blood pressure (SBP)-related AA deaths decreased globally during the study period. This is mainly due to the reduction of AA deaths attributable to high SBP in the High SDI quintile, as this proportion displayed a gentle increasing trend in all the other SDI quintiles (Figure 5A). Between 1990 and 2017, the proportions of AA deaths attributable to smoking also declined globally. Specifically, smoking-related AA deaths showed a downward trend in all SDI quintiles, most notably in the high SDI quintile (Figure 5B). Commonly, both smoking and high SBP had the most important contributions to deaths in the high-middle SDI region, and their proportion was much higher than other regions for a long period of time.

**Figure 6:**
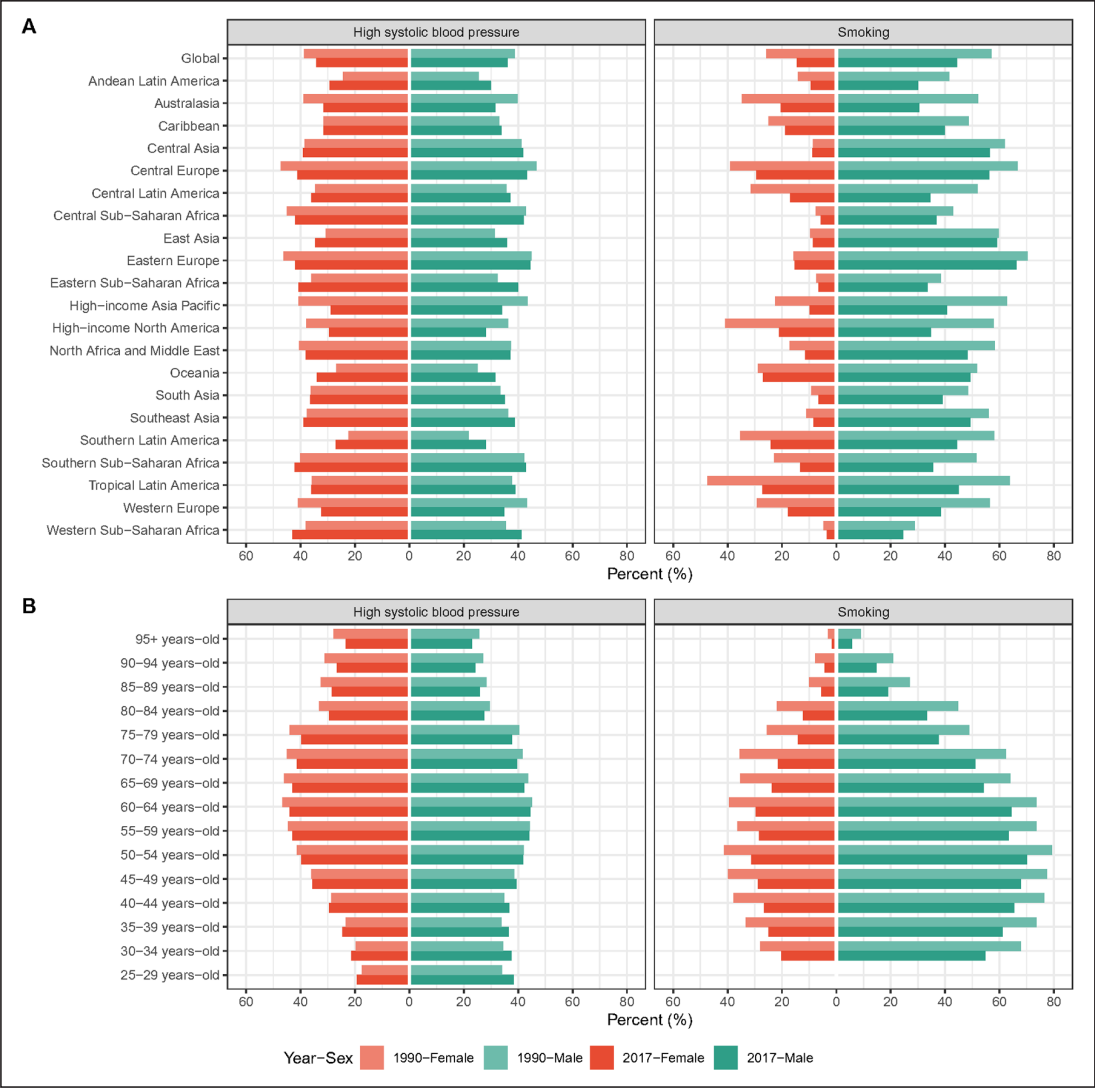
The proportion of deaths attributable to high systolic blood pressure/smoking (**A**) globally and in 21 GBD regions and (**B**) in different age groups in 1990 and 2017.

With regard to risk factors, there were many heterogeneities in different geographic locations, ages, sexes, and variations between years. For 21 GBD regions, AA deaths attributable to high SBP are roughly equal between sexes, while the percentage of AA deaths attributable to smoking in males is much higher than that in females. The good news is that in all GBD regions, AA deaths attributable to smoking decreased in 2017 when compared with that of 1990 in both sexes (Figure 6A). For different ages, among people under 50, the proportion of AA deaths attributable to high SBP in male is slightly higher than that in female, and for both sexes the proportion is higher in 2017 than in 1990. Among people over the age of 50, AA deaths attributable to high SBP among male and female were roughly equal, and declined slightly in 2017. At all ages, the percentage of AA deaths attributable to smoking in males is significantly higher than that in females, and we can also see a decreasing trend of AA deaths attributable to smoking in both sexes over time (Figure 6B).

## Discussion

This research thoroughly revealed the latest global burden of AA deaths and DALYs and the most relevant risk factors from 1990 to 2017. In general, the all-encompassing absolute number of AA deaths and DALYs were increased, while the overall ASRs of both of them declined. The patterns and trends of AA burden varied considerably by location, age, and sex and also differed across risk factors. We believe that our results are the most comprehensive and representative, which can serve as an important extension to previous studies and can furthermore help the design of targeted strategies in AA prevention tailored to different populations.

We indicated a substantial AA burden of 167,249 deaths and 3,039,858 DALYs worldwide in 2017. These figures even almost certainly represent underestimates of the impact of AA for inevitable reasons [11]. Consideration of local conditions is essential, and targeted health policies will likely be the key for overall success. The area with towering concentration of AA burden that requires special attention was the high SDI quintile, not only in terms of the absolute amount of deaths and DALYs, but also the ASRs of both. The 21 GBD regions were: Western Europe, South Asia, East Asia, high-income Asia Pacific, and high-income North America. AA was even reported to be the fifth leading cause of cardiovascular disease DALYs in high-income Asia Pacific [10]. At the country and territory level, Japan, India, China, and United States were the four countries with the highest reported AA deaths and DALYs; this further highlights the need for greater awareness of AA in those areas.

Understanding the temporal trends of AA burden also facilitates the initiation of more targeted public-health strategies. Contrary to the 59.58% increase in deaths and 45.49% increase in DALYs over the past 28 years, the ASDR and age-standardized DALY rate decreased, indicating that population aging and growth mostly accounted for the absolute increase in global AA burden. Regarding the analysis of SDI levels, the AA deaths and DALYs increased in all SDI quintiles, precipitously increased in the middle and the low-middle SDI quintiles and less obviously in the high SDI quintile. Correspondingly, the ASDR and the age-standardized DALY rate in the high SDI quintile were on the decline. From a regional perspective, the most pronounced increase of AA burden was observed in high-income Asia Pacific, Central Asia, and Eastern Europe, while high-income North America, Western Europe, Australasia had made substantial strides in reducing ASRs. These findings are constant with previous published studies [12,13,14,15,16] and similar patterns of epidemiological change were observed in the ASRs of overall cardiovascular diseases [10]. This phenomenon has to be taken into account for policymakers to formulate relevant policies more rationally, either increase support for AA prevention and treatment or continue to maintain the current gratifying trend, respectively.

We also validated that the amplitude of ASR variations from 1990 to 2017, namely EAPC, was significantly negatively correlated with baseline ASR in 1990. For those countries and territories with higher ASR in 1990, the AA burden was more likely to decrease. One possible explanation is that countries and territories with higher ASRs are also more likely to bear a heavier burden of all kinds of cardiovascular diseases. Significant public health efforts, such as improved management of various cardiovascular disease risk factors, continuous disease monitoring, and prevention of complications have been made to counter this problem. Those countries are also more likely to consider AA as a high priority in disease-prevention programs. For example, UK [17], Sweden [18], America [19], and Canada [20] adopted national screening policies, which undoubtedly saves lives and alleviates the AA burden imposed on medical healthcare systems.

Furthermore, we found positive associations between EAPC and HDI in 2017 at the beginning while negative associations were observed when the HDI exceeded about 0.8 (the ‘very high’ level). The tide of AA burden appears to have been mitigated in countries with very high HDIs. The favorable pattern of the obvious downward trend of ASRs may reflect the benefits of robust health systems that are stemming AA burden via risk factor modulation. In addition, improvements in treatment [21] and supportive nursing measures [19] in recent years have further promoted this trend [22,23]. These findings provide clear confirmation that AA prevention can no longer be a priority of only well developed areas. There are challenges ahead for those countries with high/medium HDIs where relatively enormous growth in ASRs was noticed. The health systems in these regions are insufficient to cope with a foreseeable future increase in AA burden. This may indicate the need for more active localized prevention policy interventions to tackle the diverse challenges faced by the health-care systems. Moreover, it is conceivable that the extent of the AA burden in low HDI areas was likely to be underestimated. It is essential to improve disease monitoring in these areas and promote the implementation and evaluation of relevant health policies. More targeted strategies aimed to modify multiple risk factors and improve the availability and affordability of medical care for AA are urgently needed.

The proportion of annual young deaths and DALYs remained stable. The major burden of AA is the elderly over 50 years old, accounting for more than 90% of the total. The extremely high AA burden in the high SDI quintile is also mainly because of the high proportion of elderly cases. We also noticed that the increase in AA cases in high-income Asia Pacific, East Asia, South Asia, and Eastern Europe, and the decrease in AA cases in high-income North America was dominated by the changes in AA cases among people over 50 (especially those over 70). The increase in life expectancy and the consequent rapid and ongoing increase in the elderly population in those areas in recent years should be an important reason. What’s more, it’s reported that AA are now presenting later in life [13,22]. Based on trial evidence of screening efficacy, Howard et al. suggested that older age groups should be considered in screening programs [24].

Both the absolute number and ASR of deaths/DALYs of males were considerably higher than those of females. The peak age of AA burden in males was earlier than that in females. These discrepancies partially reflected the consequence of different risk factor distributions between sexes; the protective role of estrogen, local difference in vascular hemodynamics, and many other pathophysiological factors may also contribute to the sex differences. Besides, it should not be ignored that these findings may be partly due to selection bias, in which case males were more likely to be screened for AA than their female counterparts. We also noted the global increase of AA-related deaths for both sexes. It is worth noting that females achieved a higher increase in deaths than males; simultaneously, the ASDR of males decreased more obviously. The observations associated with DALY were congruent with those observed for deaths. The sex ratios for AA burden will probably change in the future. This may be an early warning to the fact that AA may increasingly affect females like other cardiovascular diseases [12,14,25].

Many health conditions and lifestyle habits put the aortic wall at risk of damage. Among them the high SBP [26] and smoking [27] have been extensively investigated. Many of the disparities of AA burden distribution can be explained by the heterogeneity of risk factor exposures. From 1990 to 2017, the proportions of AA deaths attributable to high SBP and smoking declined, which was more pronounced in the high SDI quintile. This is largely aligned with the temporal trends of ASRs of death and DALY. Moreover, the AA deaths attributable to high SBP is roughly equal between sexes, while the percentage of AA deaths attributable to smoking in males is much higher than that in females. This is also consistent with the male bias in AA death/DALY cases and ASRs. Therefore, more aggressive preventive interventions are needed, with emphasis on smoking prevention or cessation and blood pressure control, in order to maintain the downward trend of ASRs.

Owing to the widespread use of antihypertensive medications, global mean blood pressure has remained constant or has decreased slightly over the past four decades. In contrast, the global prevalence of hypertension has increased, while the proportions of hypertension awareness, treatment and blood pressure control were low [28]. Among them, countries and territories in the low SDI, low-middle SDI, and middle SDI quintiles were particularly lagging behind and their contribution to AA deaths was increasing. It is urgent to strengthen blood pressure management to correct this trend.

It is promising that the proportion of AA deaths attributable to smoking has been decreasing over the last couple of decades. The decline might be primarily attributable to the comprehensive anti-tobacco policies [22]. Large reductions in the estimated age-standardized prevalence of daily smoking were observed at the global level, especially pronounced in Australia, Brazil, China, Norway, Sweden, Switzerland, and United States, suggesting sustained progress in tobacco control [29]. Notably, there was a possible dose-response relationship between smoking and AA deaths [26], which confirmed this conjecture. Despite the great success of years of anti-tobacco efforts, smoking remains the leading risk factor of AA burden, and the pace of progress in reducing smoking prevalence has been heterogeneous. As more countries begin to recognize the enormous preventable tobacco-induced AA burden of death and disability, and the desire of most smokers to quit once they become aware of the risks of tobacco use, the goal of a tobacco-free world by 2040 will become more feasible [30].

Apart from high SBP and smoking, several other factors are also important in determining the risk of AA burden, including elevated cholesterol and triglycerides, sedentary lifestyle, obesity, a past history of arterial aneurysms in other blood vessels, family history of aneurysms, bicuspid aortic valve, and a history of chronic inflammatory disease. Atherosclerosis shares many risk factors with abdominal AA and is strongly associated with its development, while degenerative changes most often cause thoracic AA. From Figure 5 we can see that smoking and high SBP have always been the most important contributors in AA deaths. Their total contribution has been declining year by year, from 85.23% to 68.99%, which strongly reminds us to pay more attention to the other AA risk factors that have not yet attracted enough attention. We hope that in the future, more far-ranging epidemiological investigations can also pay attention to these risk factors to facilitate a comprehensive analysis of the attribution of AA burden.

Health policymakers need timely and accurate information on the AA burden to assess the effectiveness of current related policies and allocate limited resources. Although the GBD study 2017 provided high-quality estimates of global AA burden, several limitations affected the present investigation. Differences in data collection and the quality of data sources were still inevitable. In many countries, reporting bias due to the scarcity or inferior quality of existing data were likely significant. The true AA-related deaths might be underestimated, especially in the low-SDI area, where imaging examinations were less routine and autopsy were rarely performed. For these countries and territories, results mainly relied on covariates known to be associated with AA, trends in neighboring countries, or a combination of both methods. It was even possible that in regions and countries where the burden of AA seems to be increasing partly due to improved diagnosis and reporting. Another limitation is represented by the lack of data concerning other AA-related risk factors besides high SBP and smoking. They were not available in the GBD 2017 datasets and, as such, are not accounted for.

## Conclusion

In conclusion, AA remained an important public health issue with an incremental burden globally. It was noteworthy that the changing pattern of AA burden presented a mixed picture: on the one hand, although we have achieved a reduction in ASRs in some developed areas that used to have a heavy AA burden, deaths and DALYs caused by AA remained an important health issue in those relatively underdeveloped areas and might be much more important in the near future; on the other hand, the trend that AA may increasingly affect the elderly and females cannot be ignored. Prevention of AA attributable to tobacco consumption through government policy intervention and blood pressure control should be emphasized in several high-risk areas.

## Data Accessibility Statement

All the original data we used for analysis in the present study are publicly accessible on the website of the Institute for Health Metrics and Evaluation (IHME) and can be downloaded at http://ghdx.healthdata.org/gbd-results-tool for free.

## Additional Files

The additional files for this article can be found as follows:

10.5334/gh.920.s1 Supplementary file 1. Detailed methods.

10.5334/gh.920.s2 Supplementary file 2. e-Figures.

10.5334/gh.920.s3 Supplementary file 3. e-Tables.
