# Supplementary material for: Global Burden of Aortic Aneurysm and Attributable Risk Factors from 1990 to 2017
Source: Glob Heart. 2021 May 4;16(1):35. doi: 10.5334/gh.920 (PMC8103850; doi:10.5334/gh.920)
Supplement: Supplementary file 1. — Detailed methods. [file gh-16-1-920-s1.pdf]

Methods

Study data

Information on annual AA deaths, DALYs, and respective age-standardized rate (ASR), by locations, ages, and sexes, from 1990 to 2017, were retrieved from the Global Health Data Exchange (GHDx) query tool (http://ghdx.healthdata.org/gbd-results-tool)[9]. The general methods for the GBD study and for the estimation of AA burden have been detailed in previous studies[8, 10]. e-Table 1 delineates the subgroups of the International-Classification-of-Diseases-10 codes that embody the GBD AA cause code (B.2.8).

To further analyze the global AA burden distribution, we classified the location information into three levels. Firstly, we used the social-demographic-index (SDI), which is based on national-level income per capita, average years of education among persons older than 15, and total fertility rate, to categorize the countries and territories into five SDI quintiles (High, High-middle, Middle, Low-middle, and Low). Secondly, as shown in Table 1 and 2, the world was geographically divided into 21 GBD regions. Lastly, we showed AA burden in 195 countries and territories by drawing world maps. The human-development-index (HDI) serves as a summary measure of average achievement in key dimensions of human development: a long and healthy life, being knowledgeable and have a decent standard of living. We also collected the 2017 HDI data for each country from the World Bank.

Attributable AA burden

The proportion data of AA burden attributable to different risk factors were also collected from the GBD study 2017 datasets. The general methods have been detailed by the GBD 2017

Risk Factor Collaborators in Lancet[5]. Briefly, they used a comparative risk assessment framework and included 476 risk–outcome pairs that met the GBD study criteria for convincing or probable evidence of causation. Relative risk and exposure estimates were extracted from 46 749 randomized controlled trials, cohort studies, household surveys, census data, satellite data, and other sources. They used statistical models to pool data, adjust for bias, incorporate covariates, and estimated the portion of deaths and DALYs that could be attributed to a given risk by using the counterfactual scenario of theoretical minimum risk exposure level.

Statistical analysis

We used ASRs [age-standardized death rate (ASDR) and age-standardized DALY rate] to determine the burden of AA. Standardization is necessary when comparing populations with different age structures or for the same population over time in which the age profiles change accordingly. ASRs (per 100,000 population) is equal to the sum of the product of the specific age ratio (a_i) in age group i and the number (or weight) (w_i) of the selected reference standard population group i divided by the sum of number (or weight) of the standard population, i.e.$ASR = \frac{\sum_{i=1}^{A} a_i w_i}{\sum_{i=1}^{A} w_i} \times 100{,}000$. More importantly, the ASR trends can serve as a good surrogate for shifting patterns of disease within a population, and the estimated-annual-percentage-change (EAPC) is a widely used measure of the ASR trend over a specified interval. Consequently, a regression line was fitted to the natural logarithm of the rates: $y = \alpha + \beta x + \varepsilon$, where y represents $\ln ASR$ and x refers to the calendar year. $EAPC = 100 \times (exp(\beta) - 1)$ and its 95% uncertainty interval (UI) can also be obtained from the regression model. If the EAPC estimation and its lower limit of 95% UI are both

positive, the ASR is considered to be in an increasing trend. Conversely, if the EAPC estimation and its upper limit of 95% UI are both negative, the ASR is in a downward trend. If the above conditions are not met or the estimated UI overlap, the ASR is deemed to be stable. Additionally, to explore the influential factors for EAPCs, we assessed the association between EAPCs and ASRs (1990)/HDI (2017) at the national level. All statistical analyses were performed using R program (Version 3.6.3, R core team). A *p* value of less than 0.05 was considered statistically significant.
