# Supplementary material for: Global Burden of Aortic Aneurysm and Attributable Risk Factors from 1990 to 2017
Source: Glob Heart. 2021 May 4;16(1):35. doi: 10.5334/gh.920 (PMC8103850; doi:10.5334/gh.920)
Supplement: Supplementary file 2. — e-Figures. [file gh-16-1-920-s2.pdf]

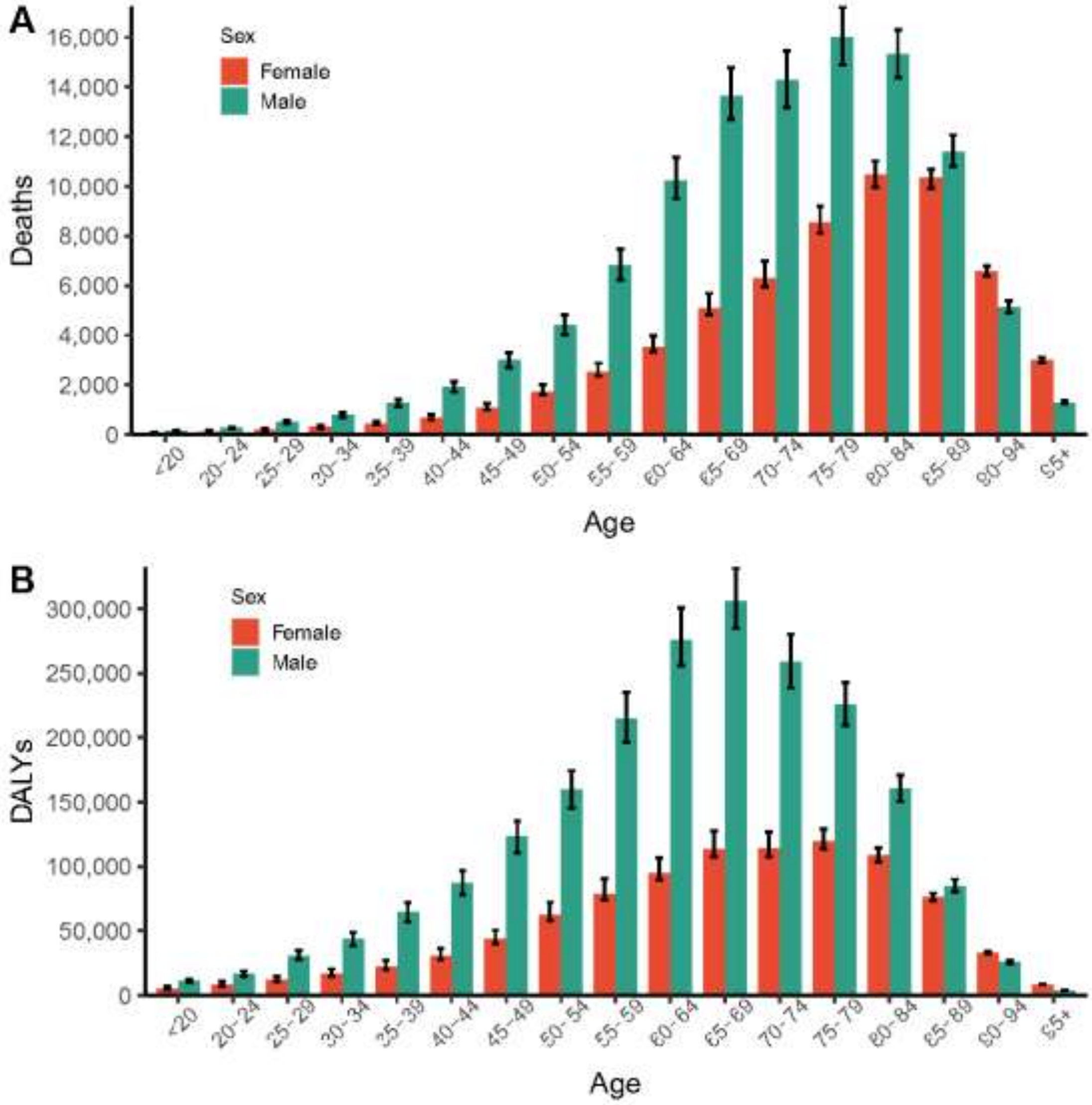

e-Figure 1. The global aortic aneurysm burden in both sexes and different age groups in 2017. A. The absolute number of death cases. B. The absolute number of DALYs. DALY, disability-adjusted life year

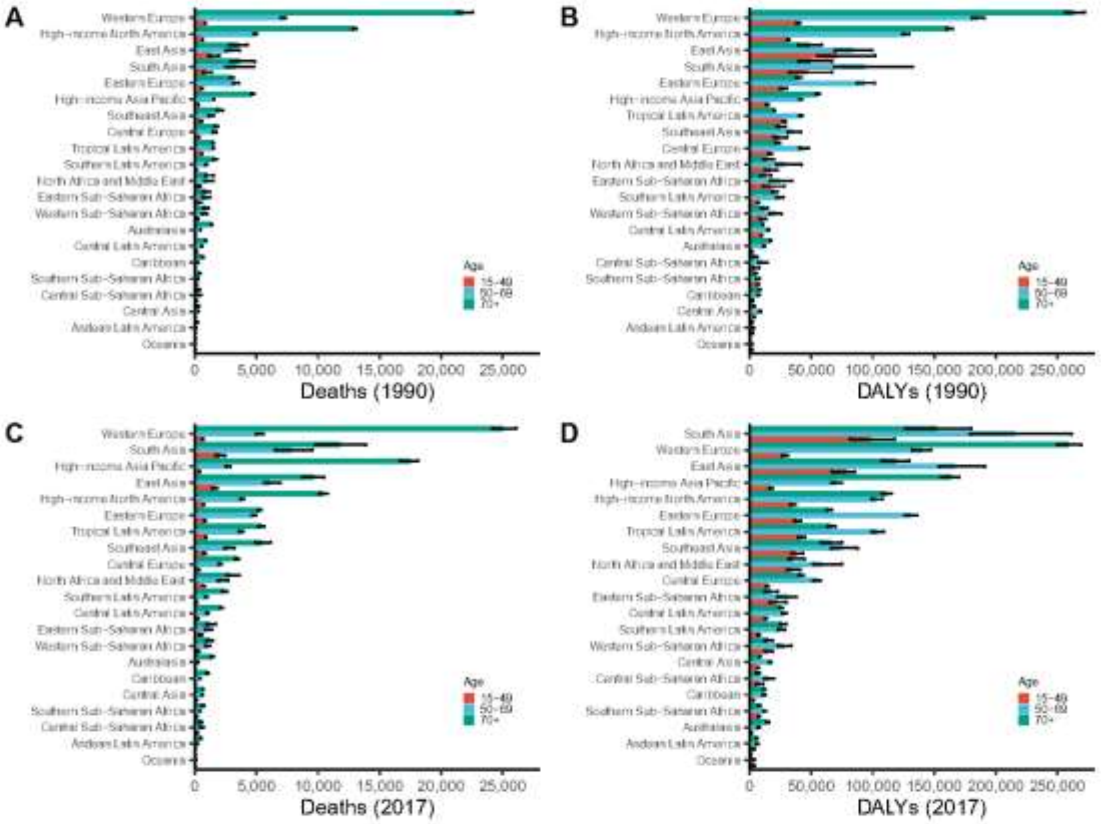

e-Figure 2. The proportion of the three age groups (15-49 years, 50-69 years and 70+ years) for aortic aneurysm deaths in 21 GBD regions between 1990 and 2017. GBD: global burden of disease.

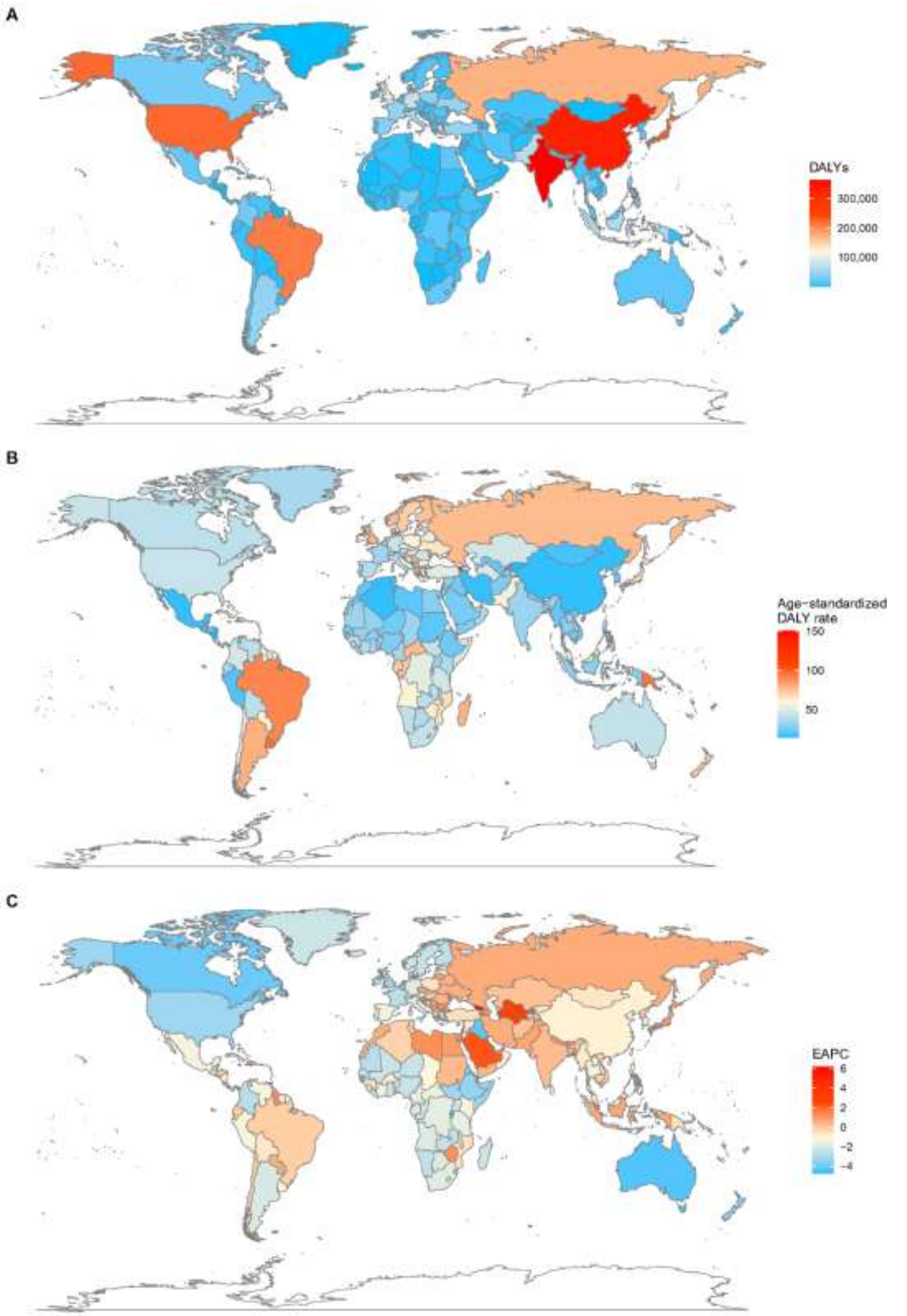

e-Figure 3. The global DALYs burden of AA in 195 countries and territories. A. The absolute number of AA DALYs in 2017. B. The AA age standardized DALY rate (per 100,000 persons) in 2017. C. The EAPC of AA age standardized DALY rate between 1990 and 2017. AA, aortic aneurysm; EAPC, estimated annual percentage change.

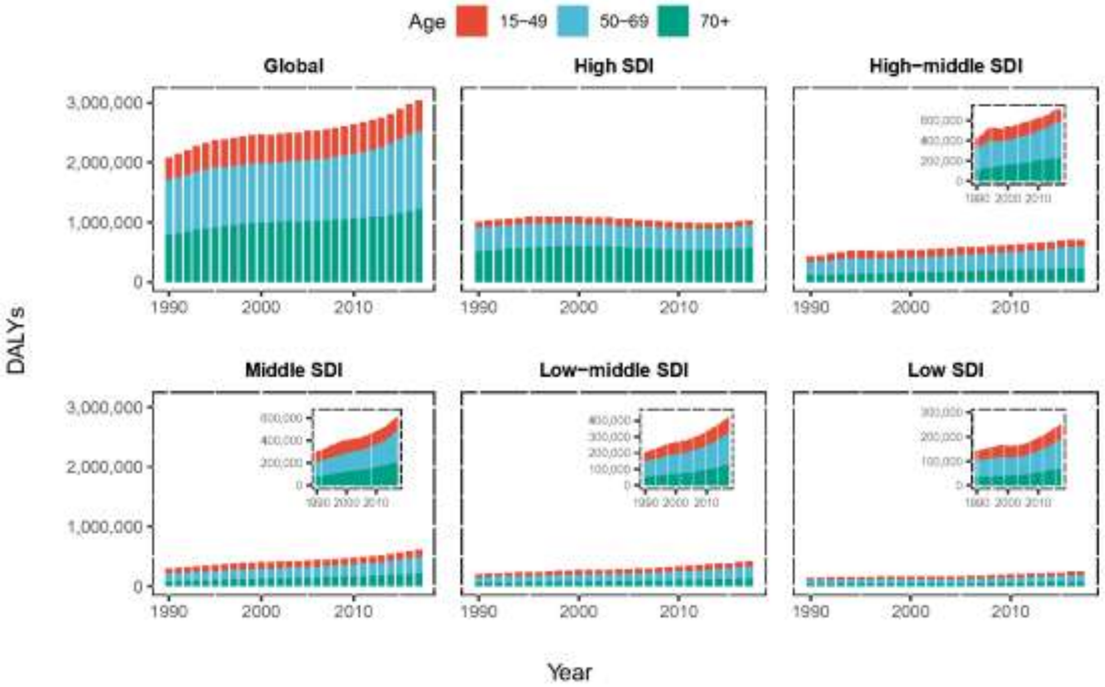

e-Figure 4. The proportion of the three age groups (15-49 years, 50-69 years and 70+ years) for aortic aneurysm DALYs globally and in 5 SDI quintiles between 1990 and 2017. DALYs, disability-adjusted life years; SDI: social-demographic index.
