# Supplementary material for: Global Burden of Aortic Aneurysm and Attributable Risk Factors from 1990 to 2017
Source: Glob Heart. 2021 May 4;16(1):35. doi: 10.5334/gh.920 (PMC8103850; doi:10.5334/gh.920)
Supplement: Supplementary file 3. — e-Tables. [file gh-16-1-920-s3.pdf]

eTable 1 ICD-10 code and subgroups of GBD cause code B.2.8 (Aortic aneurysm).

Code	Disease
**I71**	Aortic aneurysm and dissection
**I71.0**	Dissection of aorta
**I71.00**	Dissection of unspecified site of aorta
**I71.01**	Dissection of thoracic aorta
**I71.02**	Dissection of abdominal aorta
**I71.03**	Dissection of thoracoabdominal aorta
**I71.1**	Thoracic aortic aneurysm, ruptured
**I71.2**	Thoracic aortic aneurysm, without rupture
**I71.3**	Abdominal aortic aneurysm, ruptured
**I71.4**	Abdominal aortic aneurysm, without rupture
**I71.5**	Thoracoabdominal aortic aneurysm, ruptured
**I71.6**	Thoracoabdominal aortic aneurysm, without rupture
**I71.8**	Aortic aneurysm of unspecified site, ruptured
**I71.9**	Aortic aneurysm of unspecified site, without rupture

GBD, global burden of disease; ICD-10, International Classification of Disease 10.

e-Table 2 The death cases and age-standardized death rate of aortic aneurysm in 1990 and 2017, and its temporal trends from 1990 to 2017 in 195 countries and territories.

Countries and territories	1990		2017		1990-2017
	Death cases	ASR per 100,000	Death cases	ASR per 100,000	EAPC
	No. $\times 10^3$ (95% UI)	No. (95% UI)	No. $\times 10^3$ (95% UI)	No. (95% UI)	No. (95% UI)
Afghanistan	79.03(37.51 - 167.81)	1.23 (0.63 - 2.6)	119.88 (77.88-208.59)	1.22 (0.79-2.06)	-0.02 (-0.09 to 0.04)
Albania	21.23(18.95 - 25.26)	1 (0.89 - 1.2)	43.66 (34.68-53.92)	1.07 (0.85-1.32)	0.41 (0.12 to 0.71)
Algeria	106.05(75.34 - 150.35)	0.96 (0.68 - 1.35)	280.75 (202.9-382.45)	0.93 (0.68-1.27)	-0.06 (-0.14 to 0.02)
American Samoa	0.73(0.61 - 0.82)	4.02 (3.41 - 4.61)	0.81 (0.7-0.98)	2.42 (2.12-2.85)	-2.19 (-2.55 to -1.83)
Andorra	4.14(3.06 - 5.33)	7.58 (5.69 - 9.64)	6.99 (5.12-8.9)	4.78 (3.46-6.15)	-1.99 (-2.15 to -1.83)
Angola	147.17(91.78 - 229.61)	4.58 (3.08 - 6.92)	267.65 (209.36-344.66)	3.14 (2.47-3.98)	-1.81 (-1.99 to -1.62)
Antigua and Barbuda	1.47(1.31 - 1.63)	2.7 (2.43 - 2.99)	1.93 (1.76-2.14)	2.02 (1.83-2.24)	-1.46 (-1.69 to -1.22)
Argentina	2150.36(1947.55 - 2418.52)	6.83 (6.21 - 7.67)	2565.24 (2311.44-2871.44)	4.64 (4.18-5.19)	-1.78 (-1.97 to -1.58)
Armenia	156.96(136.81 - 181.83)	6.38 (5.51 - 7.39)	340.95 (316.95-365.78)	8.26 (7.71-8.87)	1.03 (0.84 to 1.22)
Australia	1447.62(1387.93 - 1501.94)	7.14 (6.86 - 7.39)	1316.73 (1184.95-1453.28)	2.89 (2.6-3.2)	-4 (-4.23 to -3.77)
Austria	440.53(420.46 - 464.64)	3.54 (3.39 - 3.73)	446.33 (409.87-485.54)	2.33 (2.14-2.52)	-2.17 (-2.43 to -1.91)
Azerbaijan	59.37(46.67 - 80.21)	1.22 (0.93 - 1.71)	154.25 (126.56-179.74)	2.02 (1.65-2.36)	1.34 (1.09 to 1.59)
Bahrain	2.35(2.03 - 3)	1.5 (1.26 - 2.02)	6.67 (5.7-7.83)	0.95 (0.82-1.14)	-2.01 (-2.26 to -1.77)
Bangladesh	638.87(446.6 - 922.83)	1.51 (1.09 - 2.18)	1686.41 (1323.02-2174.22)	1.59 (1.25-2.06)	0.44 (0.27 to 0.61)
Barbados	9.35(8.22 - 10.63)	2.87 (2.54 - 3.23)	11.84 (10.59-13.3)	2.38 (2.13-2.67)	-1.1 (-1.29 to -0.91)
Belarus	276.17(226.25 - 318.46)	2.1 (1.72 - 2.42)	398.62 (358.94-440.78)	2.48 (2.24-2.75)	0.5 (0.28 to 0.71)
Belgium	791.25(748.14 - 835)	4.82 (4.57 - 5.07)	752.13 (688.8-816.84)	2.82 (2.59-3.07)	-2.47 (-2.69 to -2.26)
Belize	1.58(1.4 - 1.86)	1.73 (1.53 - 2.04)	3.92 (3.47-4.27)	1.68 (1.49-1.84)	-0.8 (-1.24 to -0.35)
Benin	45.9(34.59 - 60.51)	2.45 (1.85 - 3.2)	67.99 (48.92-92.76)	1.69 (1.24-2.28)	-1.6 (-1.77 to -1.43)
Bermuda	4.93(4.04 - 5.75)	8.13 (6.7 - 9.46)	6.7 (6.06-7.34)	5.1 (4.61-5.59)	-1.94 (-2.09 to -1.79)
Bhutan	4.09(2.91 - 5.73)	1.95 (1.42 - 2.69)	13.39 (10.11-17.81)	2.53 (1.92-3.35)	1.1 (1.04 to 1.15)
Bolivia	73.06(42.45 - 102.6)	2.49 (1.5 - 3.46)	175.94 (132.63-228.06)	2.25 (1.71-2.91)	-0.35 (-0.4 to -0.31)
Bosnia and Herzegovina	96.96(80.73 - 116.96)	2.54 (2.15 - 3.05)	181.02 (140.62-205.94)	3.13 (2.46-3.53)	0.77 (0.56 to 0.97)

Countries and territories	1990		2017		1990-2017
	Death cases	ASR per 100,000	Death cases	ASR per 100,000	EAPC
	No. $\times 10^3$ (95% UI)	No. (95% UI)	No. $\times 10^3$ (95% UI)	No. (95% UI)	No. (95% UI)
Botswana	16.48(12.98 - 20.26)	3.34 (2.67 - 4.04)	25.79 (20.58-32.95)	2.35 (1.87-2.97)	-1.45 (-1.62 to -1.28)
Brazil	3485.88(3406.06 - 3575.59)	4.09 (4 - 4.19)	9921.64 (9459.61-10303.31)	4.49 (4.29-4.67)	0.11 (-0.03 to 0.25)
Brunei	5.53(4.36 - 6.8)	6.27 (5.13 - 7.58)	13.18 (11.78-14.81)	5.27 (4.7-5.93)	-0.55 (-0.72 to -0.39)
Bulgaria	186.08(172.74 - 198.74)	1.57 (1.46 - 1.67)	292.19 (268.12-316.38)	2.15 (1.98-2.32)	1.32 (1.07 to 1.57)
Burkina Faso	74.47(52.5 - 99.35)	1.93 (1.37 - 2.56)	120.63 (89.9-162.29)	1.62 (1.21-2.14)	-0.67 (-0.75 to -0.59)
Burundi	91.77(63.72 - 132.14)	4.8 (3.38 - 6.69)	65.7 (45.42-92.3)	1.99 (1.37-2.77)	-3.95 (-4.34 to -3.57)
Cambodia	50.62(30.4 - 81.55)	1.36 (0.87 - 2.15)	132.27 (109.35-170.42)	1.45 (1.19-1.84)	0.18 (0.12 to 0.24)
Cameroon	101.99(78.08 - 132.21)	2.71 (2.09 - 3.46)	178.32 (137.18-230.79)	1.82 (1.41-2.35)	-1.73 (-1.87 to -1.58)
Canada	2007.32(1933.23 - 2087.59)	5.92 (5.71 - 6.15)	1909.92 (1757.84-2074.63)	2.62 (2.41-2.86)	-3.84 (-4.16 to -3.52)
Cape Verde	2.43(1.94 - 2.99)	1.02 (0.81 - 1.25)	5.76 (4.86-6.66)	1.29 (1.08-1.5)	0.76 (0.65 to 0.86)
Central African Republic	49(26.56 - 77.82)	5.05 (3.06 - 7.76)	63.86 (37.56-97.6)	3.57 (2.36-5.24)	-1.56 (-1.68 to -1.44)
Chad	57.61(41.76 - 74.88)	2.18 (1.58 - 2.81)	79.62 (58.47-106.9)	1.71 (1.27-2.26)	-1.13 (-1.28 to -0.97)
Chile	277.9(261.61 - 295.9)	2.88 (2.72 - 3.06)	649.92 (581.05-722.14)	2.8 (2.5-3.11)	-0.08 (-0.34 to 0.19)
China	7513.14(6059.76 - 9199.88)	0.98 (0.78 - 1.17)	15759.96 (14248.2-17571.03)	0.88 (0.79-0.97)	-0.7 (-0.87 to -0.53)
Colombia	644.17(616.29 - 677.45)	3.85 (3.68 - 4.06)	1352.38 (1181.06-1534.02)	2.51 (2.19-2.85)	-2.45 (-2.83 to -2.06)
Comoros	8.52(5.68 - 12.37)	4.74 (3.14 - 6.77)	9.01 (6.37-11.91)	2.29 (1.62-3.01)	-3.19 (-3.57 to -2.8)
Congo	53.42(37.9 - 77.68)	5.57 (4.15 - 7.83)	76.27 (57.86-94.63)	3.81 (2.9-4.7)	-1.83 (-2.07 to -1.59)
Costa Rica	34.45(30.16 - 39.72)	2.02 (1.76 - 2.34)	126.63 (114.03-141.45)	2.62 (2.36-2.92)	0.72 (0.53 to 0.92)
Cote d'Ivoire	88.53(63.74 - 111.54)	2.59 (1.89 - 3.24)	167.89 (130.88-210.74)	1.95 (1.56-2.42)	-1.35 (-1.5 to -1.19)
Croatia	122.02(115.24 - 129.34)	1.92 (1.82 - 2.03)	313.81 (289.44-339)	3.47 (3.21-3.74)	2.54 (2.17 to 2.9)
Cuba	547.41(493.24 - 615.66)	5.26 (4.75 - 5.92)	741.28 (658.4-837.68)	3.82 (3.39-4.32)	-1.48 (-1.63 to -1.34)
Cyprus	55.89(44.21 - 72.91)	6.61 (5.31 - 8.55)	79.14 (66.33-102.57)	4.09 (3.45-5.24)	-2.2 (-2.61 to -1.79)
Czech Republic	299.15(282.97 - 315.81)	2.16 (2.05 - 2.27)	580.4 (540.35-626.03)	2.75 (2.56-2.96)	1.34 (1.08 to 1.6)
Democratic Republic of the Congo	488.75(325.78 - 701.08)	3.84 (2.53 - 5.38)	734.37 (537.67-970.4)	2.62 (1.93-3.41)	-1.82 (-2.08 to -1.55)
Denmark	601.9(556.44 - 648.59)	6.8 (6.3 - 7.3)	603.71 (551.87-656.46)	4.91 (4.49-5.34)	-1.52 (-1.79 to -1.26)

Countries and territories	1990		2017		1990-2017
	Death cases	ASR per 100,000	Death cases	ASR per 100,000	EAPC
	No. $\times 10^3$ (95% UI)	No. (95% UI)	No. $\times 10^3$ (95% UI)	No. (95% UI)	No. (95% UI)
Djibouti	4.97(3.09 - 7.38)	4.05 (2.58 - 5.77)	10.83 (6.97-15.33)	2.43 (1.58-3.39)	-2.6 (-2.95 to -2.25)
Dominica	3.62(3.15 - 4.09)	4.65 (4.07 - 5.23)	4.28 (3.86-4.72)	4.37 (3.94-4.81)	-0.48 (-0.6 to -0.37)
Dominican Republic	59.76(50.5 - 68.54)	1.7 (1.44 - 1.97)	181.08 (151.41-221.7)	2.02 (1.69-2.48)	0.72 (0.43 to 1.02)
Ecuador	101.05(88.14 - 113.9)	1.96 (1.71 - 2.21)	280.08 (253.23-313.19)	1.97 (1.78-2.21)	0.05 (-0.13 to 0.24)
Egypt	267.88(170.14 - 489.82)	1.12 (0.74 - 2.03)	709.93 (415.1-1156.24)	1.44 (0.88-2.32)	0.85 (0.77 to 0.93)
El Salvador	35.47(30.71 - 40.56)	1.23 (1.06 - 1.41)	75.81 (62.56-89.61)	1.3 (1.07-1.54)	0.27 (0.09 to 0.46)
Equatorial Guinea	8.81(5.04 - 14.46)	5.15 (3.23 - 8.18)	12.06 (8.34-16.83)	3.21 (2.27-4.37)	-2.26 (-2.56 to -1.95)
Eritrea	42.78(25.74 - 70.42)	5.17 (3.45 - 8.05)	46.83 (29.82-64.76)	2.47 (1.62-3.34)	-3.38 (-3.67 to -3.09)
Estonia	57.69(52.94 - 62.89)	2.75 (2.53 - 2.98)	77.35 (65.67-96.02)	2.81 (2.38-3.46)	-0.07 (-0.42 to 0.28)
Ethiopia	425.53(243.69 - 725.98)	2.66 (1.7 - 4.24)	445.65 (305.2-616.19)	1.34 (0.91-1.91)	-3 (-3.22 to -2.78)
Federated States of Micronesia	2.22(1.68 - 2.81)	5.15 (4.04 - 6.41)	2.28 (1.78-2.83)	4.12 (3.41-5.02)	-1.06 (-1.15 to -0.96)
Fiji	18(15.42 - 20.73)	5.98 (5.13 - 6.92)	33.63 (28.92-38.66)	5.76 (4.98-6.56)	-0.37 (-0.58 to -0.16)
Finland	600.81(542.59 - 665.59)	8.09 (7.33 - 8.94)	582.78 (534.59-641.66)	4.44 (4.07-4.88)	-2.26 (-2.38 to -2.13)
France	3351.27(3202.83 - 3513.72)	3.72 (3.57 - 3.89)	3370.62 (3103.87-3647.59)	2.08 (1.91-2.27)	-2.65 (-2.95 to -2.35)
Gabon	27.43(20.52 - 35.36)	5.29 (3.93 - 6.72)	33.5 (25.17-40.34)	3.67 (2.76-4.45)	-1.67 (-1.81 to -1.53)
Georgia	57.22(51.23 - 64.67)	0.96 (0.87 - 1.08)	174.09 (158.01-193.84)	2.97 (2.71-3.28)	5.94 (4.94 to 6.96)
Germany	5346.26(4939.95 - 5797.64)	3.94 (3.67 - 4.24)	5233.65 (4677.69-5865.5)	2.5 (2.24-2.79)	-1.87 (-2.02 to -1.71)
Ghana	136.16(110.26 - 161.54)	2.49 (2.03 - 2.94)	232.14 (190.37-282.26)	1.75 (1.45-2.11)	-2.23 (-2.88 to -1.58)
Greece	620.18(591.53 - 651.57)	4.06 (3.88 - 4.25)	1095.14 (1005.23-1191.06)	4.23 (3.92-4.6)	-0.03 (-0.34 to 0.28)
Greenland	0.94(0.75 - 1.09)	3.5 (2.82 - 4.08)	1.24 (1.02-1.42)	2.19 (1.83-2.51)	-2.08 (-2.28 to -1.89)
Grenada	4.14(3.59 - 4.77)	5.61 (4.85 - 6.44)	7.82 (7.1-8.57)	4.62 (4.21-5.04)	-0.77 (-1.06 to -0.48)
Guam	4.23(3.08 - 5.1)	6.31 (4.71 - 7.44)	6.01 (5-8.42)	3.61 (3.03-5.02)	-2.56 (-2.82 to -2.3)
Guatemala	51.55(44.66 - 58.87)	1.77 (1.5 - 2.01)	109.55 (97.44-123.57)	1.12 (1-1.25)	-2.33 (-2.66 to -2.01)
Guinea	73.89(56.38 - 101.36)	2.42 (1.85 - 3.29)	98.99 (71.04-135.25)	2.03 (1.48-2.74)	-0.74 (-0.98 to -0.49)
Guinea-Bissau	13.78(9.06 - 19.95)	3.89 (2.67 - 5.52)	14.15 (9.43-20.11)	2.34 (1.63-3.23)	-2.18 (-2.39 to -1.98)

Countries and territories	1990		2017		1990-2017
	Death cases	ASR per 100,000	Death cases	ASR per 100,000	EAPC
	No. $\times 10^3$ (95% UI)	No. (95% UI)	No. $\times 10^3$ (95% UI)	No. (95% UI)	No. (95% UI)
Guyana	8.15(7.41 - 8.89)	2.3 (2.09 - 2.52)	17.31 (15.23-19.51)	3.2 (2.82-3.6)	1.17 (0.63 to 1.71)
Haiti	106.68(68.6 - 150.74)	3.94 (2.68 - 5.45)	202.14 (159.69-262.01)	3.69 (2.97-4.75)	-0.22 (-0.27 to -0.18)
Honduras	22.5(17.33 - 29.35)	1.17 (0.87 - 1.53)	67.93 (51.86-90.55)	1.25 (0.95-1.66)	0.14 (-0.03 to 0.31)
Hungary	381.21(365.99 - 396.51)	2.57 (2.47 - 2.67)	502.05 (465.95-540.17)	2.55 (2.37-2.74)	0.01 (-0.2 to 0.22)
Iceland	13.11(12.13 - 14.14)	4.3 (3.98 - 4.63)	17.82 (16.42-19.35)	3.05 (2.81-3.32)	-1.69 (-1.92 to -1.47)
India	6005.05(4477.59 - 8362.56)	1.58 (1.23 - 2.17)	16865.6 (13985.44-20194.29)	1.82 (1.51-2.17)	0.41 (0.21 to 0.61)
Indonesia	1047.49(852.81 - 1316.85)	1.37 (1.09 - 1.73)	2922.72 (2374.6-3797.26)	1.76 (1.44-2.27)	0.96 (0.9 to 1.01)
Iran	189.88(161.88 - 230.37)	0.83 (0.7 - 1.01)	612.14 (509.57-664.24)	0.97 (0.8-1.05)	0.81 (0.65 to 0.96)
Iraq	255.44(205.5 - 312.65)	3.85 (3.15 - 4.65)	278.6 (248.98-310.7)	1.46 (1.28-1.63)	-3.97 (-4.49 to -3.46)
Ireland	250.01(237.48 - 263.72)	5.76 (5.5 - 6.06)	306.48 (277.71-335.63)	4.05 (3.67-4.43)	-1.98 (-2.38 to -1.58)
Israel	116.71(107.26 - 127.84)	2.41 (2.23 - 2.62)	189.22 (170.97-207.86)	1.57 (1.41-1.73)	-2.03 (-2.21 to -1.85)
Italy	2663.11(2551.54 - 2800.66)	2.85 (2.74 - 2.99)	3864.28 (3565.27-4202)	2.35 (2.16-2.55)	-1.29 (-1.72 to -0.86)
Jamaica	36.88(33.1 - 42.91)	1.99 (1.79 - 2.33)	70.63 (58.98-83.03)	2.4 (1.99-2.83)	0.24 (-0.12 to 0.6)
Japan	5963.25(5856.39 - 6087.48)	3.65 (3.59 - 3.73)	19130 (18186.28-19865.08)	4.32 (4.1-4.52)	0.98 (0.8 to 1.15)
Jordan	26.83(18.58 - 35.6)	2.04 (1.44 - 2.73)	79.84 (66.47-102.76)	1.7 (1.42-2.19)	-1.08 (-1.29 to -0.87)
Kazakhstan	202.3(172.4 - 243.17)	1.57 (1.32 - 1.89)	320.53 (294.13-350.48)	1.93 (1.77-2.09)	0.19 (-0.25 to 0.64)
Kenya	191.67(137.11 - 246.43)	2.67 (1.89 - 3.5)	350.05 (272.48-446.94)	1.99 (1.54-2.61)	-1.45 (-1.63 to -1.26)
Kiribati	0.53(0.45 - 0.6)	1.56 (1.34 - 1.75)	0.95 (0.77-1.15)	1.61 (1.33-1.91)	0.11 (0.08 to 0.15)
Kuwait	8.14(7.56 - 8.77)	1.29 (1.18 - 1.41)	29.11 (24.94-34.42)	1.3 (1.11-1.54)	0.45 (0.02 to 0.88)
Kyrgyzstan	25.02(22.52 - 29.37)	0.86 (0.77 - 1.02)	31.77 (28.76-35.82)	0.78 (0.7-0.87)	-0.5 (-0.69 to -0.32)
Laos	40.4(27.86 - 63.52)	2.31 (1.65 - 3.57)	67.12 (53.26-89.08)	1.94 (1.55-2.57)	-0.8 (-0.86 to -0.74)
Latvia	90.15(82.08 - 99.96)	2.44 (2.23 - 2.69)	88.83 (78.49-99.7)	2.13 (1.88-2.39)	-0.77 (-1.08 to -0.45)
Lebanon	22.94(17.61 - 35.99)	1.15 (0.89 - 1.8)	62.19 (48.31-84.63)	1.12 (0.88-1.51)	0.07 (-0.04 to 0.18)
Lesotho	22.61(17.27 - 29.16)	2.64 (2.05 - 3.37)	25.99 (18.9-33.14)	2.51 (1.89-3.15)	0 (-0.12 to 0.13)
Liberia	27.57(18.53 - 36.01)	2.65 (1.78 - 3.44)	28.14 (20.14-37.11)	1.7 (1.22-2.23)	-1.87 (-2.18 to -1.56)

Countries and territories	1990		2017		1990-2017
	Death cases	ASR per 100,000	Death cases	ASR per 100,000	EAPC
	No. $\times 10^3$ (95% UI)	No. (95% UI)	No. $\times 10^3$ (95% UI)	No. (95% UI)	No. (95% UI)
Libya	14.99(11.18 - 21.88)	0.84 (0.63 - 1.23)	44.05 (33.09-60.88)	1.07 (0.8-1.47)	1.12 (0.95 to 1.28)
Lithuania	95.76(86.78 - 105.99)	2.07 (1.88 - 2.27)	151.35 (138.8-163.97)	2.6 (2.39-2.82)	0.92 (0.57 to 1.28)
Luxembourg	24.67(22.84 - 26.58)	4.35 (4.05 - 4.66)	32.64 (28.74-36.99)	3.14 (2.76-3.57)	-1.85 (-2.13 to -1.56)
Macedonia	24.86(21.37 - 34.28)	1.49 (1.29 - 2.03)	55.42 (43.38-65.66)	1.67 (1.31-1.97)	0.41 (0.24 to 0.59)
Madagascar	274.64(176.77 - 390.95)	5.63 (3.6 - 7.86)	338.73 (230.44-471.92)	3.64 (2.46-4.97)	-2.15 (-2.38 to -1.91)
Malawi	96.54(52.66 - 142.94)	2.73 (1.52 - 3.92)	139.92 (91.08-191.2)	2.03 (1.33-2.82)	-1.68 (-2.02 to -1.34)
Malaysia	346.08(276.42 - 399.76)	4.61 (3.65 - 5.33)	833.13 (685.37-949.1)	3.97 (3.24-4.55)	-0.84 (-1.04 to -0.64)
Maldives	2.26(1.39 - 3.12)	3.62 (2.49 - 4.91)	5.46 (4.69-6.25)	2.19 (1.88-2.52)	-2.38 (-2.67 to -2.09)
Mali	90.25(56.53 - 131.81)	2.72 (1.77 - 3.87)	117.44 (82.66-160.89)	1.76 (1.25-2.37)	-1.95 (-2.2 to -1.71)
Malta	12.34(11.4 - 13.35)	2.94 (2.73 - 3.16)	21.59 (19.55-23.63)	2.33 (2.11-2.54)	-1.44 (-1.7 to -1.18)
Marshall Islands	0.84(0.52 - 1.13)	5.91 (3.89 - 7.91)	1.36 (0.89-1.82)	5.26 (3.79-6.76)	-0.6 (-0.74 to -0.46)
Mauritania	30.28(24.04 - 41.35)	3.18 (2.56 - 4.28)	32.46 (25.17-42.33)	1.87 (1.46-2.41)	-2.31 (-2.6 to -2.02)
Mauritius	10.74(9.92 - 11.64)	1.59 (1.47 - 1.72)	16.16 (14.73-17.71)	1.09 (0.99-1.19)	-1.55 (-1.75 to -1.34)
Mexico	526.84(512.54 - 543.58)	1.31 (1.27 - 1.35)	1027.43 (978.77-1071.29)	0.96 (0.91-1)	-1.36 (-1.53 to -1.2)
Moldova	42.68(38.87 - 47.51)	1.01 (0.93 - 1.11)	71 (65.53-76.57)	1.26 (1.17-1.36)	1.09 (0.76 to 1.41)
Mongolia	9.79(8.44 - 12.53)	1.02 (0.88 - 1.28)	17.45 (15.01-20.66)	0.94 (0.81-1.09)	-0.71 (-1 to -0.41)
Montenegro	40.96(36.24 - 48.65)	6.66 (5.94 - 7.93)	72.45 (63.46-83.52)	7.31 (6.43-8.4)	0.5 (0.39 to 0.62)
Morocco	125.3(87.85 - 187.69)	0.93 (0.64 - 1.39)	327.22 (240.58-457.09)	1.13 (0.84-1.58)	0.71 (0.6 to 0.82)
Mozambique	208(123.74 - 302.53)	3.94 (2.33 - 5.58)	307.27 (218.19-432.92)	3.19 (2.26-4.38)	-0.84 (-0.97 to -0.71)
Myanmar	280.81(183.84 - 410.92)	1.46 (1.01 - 2.08)	491.9 (392.22-615.04)	1.29 (1.04-1.6)	-0.53 (-0.6 to -0.47)
Namibia	23.51(17.94 - 29.28)	3.79 (2.9 - 4.62)	30.89 (24.73-37.56)	2.45 (1.98-2.94)	-2.18 (-2.6 to -1.76)
Nepal	89.72(64 - 137.6)	1.13 (0.82 - 1.71)	286.15 (223.3-369.38)	1.52 (1.19-1.95)	1.34 (1.12 to 1.56)
Netherlands	1478.5(1412.05 - 1546.43)	6.94 (6.64 - 7.25)	1480.54 (1368.81-1590.87)	4.06 (3.76-4.36)	-2.53 (-2.96 to -2.09)
New Zealand	402.91(383.27 - 422.48)	9.75 (9.29 - 10.2)	387.3 (357.79-419.86)	4.73 (4.36-5.13)	-3.28 (-3.5 to -3.06)
Nicaragua	15.72(13.22 - 18)	1.07 (0.9 - 1.23)	37.78 (33.08-44)	0.86 (0.75-1)	-1.03 (-1.17 to -0.89)

Countries and territories	1990		2017		1990-2017
	Death cases	ASR per 100,000	Death cases	ASR per 100,000	EAPC
	No. $\times 10^3$ (95% UI)	No. (95% UI)	No. $\times 10^3$ (95% UI)	No. (95% UI)	No. (95% UI)
Niger	52.6(38.14 - 70.67)	2.22 (1.63 - 2.96)	78.44 (53.69-109.99)	1.33 (0.93-1.83)	-2.22 (-2.48 to -1.97)
Nigeria	959.01(672.65 - 1317.63)	2.39 (1.71 - 3.24)	1062.76 (723.3-1653.84)	1.53 (1.06-2.33)	-2.18 (-2.42 to -1.94)
North Korea	176.94(141.57 - 218.89)	1.17 (0.94 - 1.45)	349.83 (284.82-438.6)	1.17 (0.95-1.48)	-0.2 (-0.32 to -0.09)
Northern Mariana Islands	0.71(0.58 - 0.86)	5.59 (4.6 - 6.61)	0.97 (0.84-1.19)	2.37 (2.06-2.87)	-3.72 (-3.99 to -3.45)
Norway	554.12(539.22 - 569.41)	7.35 (7.16 - 7.55)	533.35 (508.78-557.91)	5.12 (4.88-5.38)	-1.78 (-2.09 to -1.48)
Oman	14.64(10.15 - 25.24)	2.2 (1.57 - 3.7)	33.26 (23.18-49)	1.89 (1.36-2.71)	-0.56 (-0.78 to -0.34)
Pakistan	1231.08(967.53 - 1601.92)	2.36 (1.85 - 3.09)	2842.09 (2172.59-3661.63)	3.24 (2.48-4.16)	1.01 (0.93 to 1.09)
Palestine	15.23(11.7 - 20.42)	1.79 (1.39 - 2.4)	32.59 (28.37-37.45)	1.6 (1.39-1.83)	-0.36 (-0.57 to -0.14)
Panama	38.05(33.98 - 42.25)	2.64 (2.35 - 2.93)	72.35 (65.63-79.43)	1.82 (1.65-2)	-1.88 (-2.12 to -1.65)
Papua New Guinea	76.59(46.76 - 111.55)	4.71 (3.24 - 6.59)	159.65 (105.94-223.11)	4.19 (3.05-5.56)	-0.64 (-0.73 to -0.55)
Paraguay	57.86(49.25 - 65.73)	2.74 (2.35 - 3.12)	157.89 (130.47-192.28)	3.12 (2.57-3.78)	0.56 (0.4 to 0.72)
Peru	160.38(138.25 - 193.01)	1.39 (1.19 - 1.68)	333.97 (277.29-396.94)	1.07 (0.89-1.27)	-1.12 (-1.3 to -0.94)
Philippines	592.33(521.83 - 682.34)	2.28 (2.01 - 2.63)	1277.97 (1116.68-1458.54)	2.15 (1.89-2.44)	-0.97 (-1.48 to -0.46)
Poland	1489.49(1266.4 - 1747)	3.31 (2.83 - 3.88)	2045.88 (1900.34-2209.5)	2.92 (2.71-3.15)	-0.48 (-0.73 to -0.23)
Portugal	289.12(276.38 - 302.85)	2.15 (2.06 - 2.25)	468.9 (430.25-509.41)	1.79 (1.63-1.94)	-1.02 (-1.2 to -0.84)
Puerto Rico	77.83(71.52 - 84.83)	2.1 (1.93 - 2.28)	86.35 (78.65-95.55)	1.12 (1.02-1.24)	-3.14 (-3.41 to -2.86)
Qatar	2.27(1.75 - 3.45)	2.95 (2.4 - 4.27)	7.81 (6.11-9.97)	1.24 (1.02-1.55)	-4.2 (-4.68 to -3.72)
Romania	481.21(430.02 - 538.23)	1.79 (1.62 - 1.99)	770.33 (718.39-824.05)	2.08 (1.94-2.22)	0.66 (0.47 to 0.85)
Russian Federation	4751.2(4494.53 - 4973.74)	2.65 (2.51 - 2.77)	8132.14 (7871.13-8395.43)	3.49 (3.38-3.61)	0.74 (0.46 to 1.02)
Rwanda	98.47(64.13 - 146.72)	3.96 (2.55 - 5.81)	74.51 (50.37-111.9)	1.62 (1.1-2.43)	-4.25 (-4.7 to -3.79)
Saint Lucia	6.19(5.33 - 7.39)	7.05 (6.08 - 8.38)	10.78 (9.76-11.81)	5.27 (4.77-5.78)	-1.42 (-1.6 to -1.25)
Saint Vincent and the Grenadines	2.43(2.22 - 2.65)	3.24 (2.97 - 3.52)	3.92 (3.56-4.29)	2.85 (2.59-3.13)	-0.57 (-0.7 to -0.44)
Samoa	2.49(1.94 - 3.08)	3.53 (2.75 - 4.4)	3.62 (2.79-4.39)	3.14 (2.41-3.82)	-0.7 (-0.83 to -0.56)
Sao Tome and Principe	1.25(0.85 - 1.82)	1.92 (1.31 - 2.76)	1.62 (1.27-2.11)	1.78 (1.4-2.27)	-0.76 (-1.02 to -0.5)
Saudi Arabia	52.97(39.75 - 69.41)	0.95 (0.71 - 1.24)	184.48 (144.39-229.49)	1.53 (1.15-1.87)	2.44 (2.16 to 2.72)

Countries and territories	1990		2017		1990-2017
	Death cases	ASR per 100,000	Death cases	ASR per 100,000	EAPC
	No. $\times 10^3$ (95% UI)	No. (95% UI)	No. $\times 10^3$ (95% UI)	No. (95% UI)	No. (95% UI)
Senegal	61.48(45.88 - 79.91)	2.11 (1.58 - 2.71)	83.16 (66.6-103.98)	1.31 (1.06-1.64)	-2.26 (-2.59 to -1.92)
Serbia	335.2(285.72 - 395.88)	3.04 (2.62 - 3.59)	642.13 (506.58-729.39)	4.03 (3.19-4.56)	1.53 (1.37 to 1.69)
Seychelles	1.24(1.06 - 1.54)	2.2 (1.87 - 2.72)	1.91 (1.58-2.46)	1.99 (1.65-2.55)	-0.6 (-0.73 to -0.48)
Sierra Leone	47.43(34.2 - 61.6)	2.58 (1.88 - 3.33)	57.83 (45.97-72.93)	1.9 (1.52-2.38)	-1.32 (-1.47 to -1.17)
Singapore	71.8(67.35 - 76.3)	3.6 (3.38 - 3.83)	119.48 (108.43-131.6)	1.83 (1.66-2.02)	-2.55 (-2.69 to -2.42)
Slovakia	111.73(91.92 - 170.74)	1.88 (1.56 - 2.86)	166.64 (141.92-185.78)	1.88 (1.6-2.09)	0.34 (-0.03 to 0.72)
Slovenia	52.69(48.9 - 58.23)	2.16 (2.01 - 2.39)	106.26 (95.45-117.31)	2.41 (2.17-2.65)	0.17 (-0.33 to 0.67)
Solomon Islands	3.34(2.31 - 4.64)	2.92 (2.15 - 3.96)	6.52 (4.94-8.56)	2.5 (1.91-3.2)	-0.67 (-0.71 to -0.63)
Somalia	96.77(51.72 - 162.53)	4.56 (2.71 - 7.12)	126.96 (84.54-183.11)	2.41 (1.6-3.37)	-3.15 (-3.53 to -2.77)
South Africa	615.97(484.15 - 732.29)	3.1 (2.41 - 3.75)	931.88 (846.25-1110.27)	2.35 (2.14-2.81)	-1.56 (-2.26 to -0.85)
South Korea	464.83(406.67 - 535.89)	1.92 (1.7 - 2.19)	1302.29 (1172.22-1442.44)	1.63 (1.47-1.8)	-0.7 (-0.84 to -0.56)
South Sudan	85.32(47.92 - 140.97)	4.12 (2.49 - 6.4)	66.01 (42.19-98.77)	2.15 (1.39-3.13)	-3.11 (-3.45 to -2.77)
Spain	1483.47(1421.26 - 1546.74)	2.62 (2.52 - 2.73)	2472.5 (2263-2705.78)	2.27 (2.07-2.48)	-1.16 (-1.6 to -0.73)
Sri Lanka	173.14(147.43 - 206.59)	1.93 (1.67 - 2.27)	374.3 (289.72-470.86)	1.75 (1.36-2.2)	-0.6 (-0.88 to -0.33)
Sudan	86.16(55.56 - 143.01)	0.99 (0.63 - 1.63)	182.29 (120.78-279.44)	1.1 (0.72-1.71)	0.47 (0.32 to 0.62)
Suriname	6.4(5.54 - 7.23)	2.72 (2.34 - 3.08)	13.36 (11.7-15.13)	2.48 (2.17-2.8)	-0.69 (-0.89 to -0.49)
Swaziland	8.13(6.48 - 9.82)	3.32 (2.66 - 4.02)	12.49 (9.45-15.59)	2.63 (2.06-3.25)	-0.67 (-0.95 to -0.39)
Sweden	1143.78(1098.67 - 1188.37)	6.84 (6.59 - 7.09)	1059.94 (987.86-1151.52)	4.5 (4.2-4.86)	-1.97 (-2.33 to -1.6)
Switzerland	521.73(462.04 - 587.47)	4.58 (4.07 - 5.13)	545.06 (498.93-597.76)	2.8 (2.56-3.06)	-2.03 (-2.16 to -1.9)
Syria	82.58(61.67 - 116.98)	1.73 (1.34 - 2.43)	186.61 (154-229.17)	1.7 (1.41-2.06)	-0.55 (-0.79 to -0.31)
Taiwan (Province of China)	187.29(177.86 - 197.54)	1.29 (1.22 - 1.36)	996.62 (926.64-1070.01)	2.61 (2.43-2.81)	2.64 (2.21 to 3.07)
Tajikistan	43.06(35.7 - 52.57)	1.63 (1.33 - 2.01)	82.33 (68.85-91.67)	1.89 (1.53-2.14)	0.07 (-0.23 to 0.37)
Tanzania	307.8(200.73 - 418.95)	3.31 (2.17 - 4.44)	520.15 (365.03-738.63)	2.49 (1.75-3.55)	-1.7 (-2.01 to -1.39)
Thailand	607.24(479.69 - 736.62)	2.18 (1.74 - 2.63)	1627.93 (1354.47-1817.24)	1.77 (1.48-1.98)	-1.28 (-1.44 to -1.11)
The Bahamas	5.97(5.41 - 6.67)	4.14 (3.75 - 4.62)	12.21 (11.06-13.5)	3.64 (3.29-4.02)	-0.53 (-0.62 to -0.43)

Countries and territories	1990		2017		1990-2017
	Death cases	ASR per 100,000	Death cases	ASR per 100,000	EAPC
	No. $\times 10^3$ (95% UI)	No. (95% UI)	No. $\times 10^3$ (95% UI)	No. (95% UI)	No. (95% UI)
The Gambia	8.74(6.16 - 11.64)	2.82 (2.03 - 3.69)	17.14 (13.24-21.67)	2.02 (1.58-2.52)	-1.43 (-1.58 to -1.28)
Timor-Leste	2.68(1.94 - 3.74)	1.25 (0.88 - 1.75)	10.07 (7.4-16.19)	1.51 (1.13-2.35)	0.7 (0.58 to 0.82)
Togo	29.41(22.29 - 38.06)	2.75 (2.08 - 3.55)	54.67 (42.56-69.45)	1.89 (1.51-2.37)	-1.64 (-1.8 to -1.47)
Tonga	1.01(0.86 - 1.2)	2.09 (1.79 - 2.48)	1.5 (1.21-1.79)	1.98 (1.62-2.37)	-0.22 (-0.27 to -0.18)
Trinidad and Tobago	44.18(40.24 - 49.26)	5.35 (4.87 - 5.94)	72.26 (60.58-86.31)	4.18 (3.52-4.99)	-1.33 (-1.56 to -1.09)
Tunisia	41.88(31.36 - 62.58)	0.98 (0.74 - 1.48)	116.36 (82.23-164.22)	1.04 (0.74-1.46)	-0.01 (-0.14 to 0.11)
Turkey	953.47(693.94 - 1327.05)	2.8 (2.06 - 3.98)	2155.73 (1876.13-2696.34)	2.51 (2.19-3.15)	-0.35 (-0.71 to 0)
Turkmenistan	20.71(18.33 - 24.15)	1.18 (1.01 - 1.4)	80.78 (67.02-98.63)	2.36 (1.91-2.94)	2.84 (2.43 to 3.24)
Uganda	145.16(93.34 - 210.24)	2.57 (1.63 - 3.8)	196.53 (129.98-286.12)	1.72 (1.14-2.55)	-2.16 (-2.5 to -1.83)
Ukraine	1577.05(1431 - 1797.39)	2.16 (1.97 - 2.44)	1937.21 (1810.88-2076.27)	2.56 (2.38-2.73)	0.21 (-0.13 to 0.55)
United Arab Emirates	6.22(4.01 - 10.94)	1.47 (0.87 - 2.7)	67.68 (45.68-117.12)	2.07 (1.37-3.64)	1.31 (1.11 to 1.51)
United Kingdom	9399.59(9249.31 - 9543.37)	9.29 (9.15 - 9.44)	7709.94 (7516.57-7934.43)	5.37 (5.24-5.53)	-2.73 (-3.11 to -2.35)
United States	16528.93(16278.95 - 16778.96)	4.78 (4.71 - 4.86)	13081.38 (12627.95-13516.43)	2.32 (2.23-2.39)	-3.42 (-3.71 to -3.13)
Uruguay	240(221.31 - 259.02)	5.97 (5.51 - 6.42)	284.26 (253.51-316.94)	4.97 (4.41-5.56)	-0.94 (-1.09 to -0.78)
Uzbekistan	63.94(47.82 - 79.75)	0.59 (0.43 - 0.74)	219.29 (193.76-248.76)	1.3 (1.15-1.46)	3.45 (3.19 to 3.71)
Vanuatu	2.61(1.87 - 3.48)	4.41 (3.19 - 5.78)	5.9 (4.11-8.8)	4.1 (2.88-5.95)	-0.39 (-0.44 to -0.33)
Venezuela	246.1(221.72 - 273.37)	2.72 (2.46 - 3.04)	588.28 (504.41-684.51)	2.3 (1.97-2.67)	-1.1 (-1.32 to -0.89)
Vietnam	536.29(419.63 - 719.59)	1.45 (1.13 - 1.94)	1225.2 (1014.99-1505.78)	1.51 (1.26-1.85)	-0.05 (-0.16 to 0.06)
Virgin Islands, U.S.	2.66(2.36 - 2.96)	3.56 (3.19 - 3.97)	5.98 (4.89-6.89)	3.26 (2.7-3.71)	-0.48 (-0.66 to -0.3)
Yemen	50.24(27.89 - 100.07)	1.07 (0.62 - 2.07)	151.41 (104.24-224.27)	1.32 (0.91-1.92)	0.75 (0.67 to 0.83)
Zambia	79.36(53.06 - 109.91)	3.28 (2.15 - 4.41)	115.05 (81.38-146.81)	2.13 (1.52-2.73)	-2.45 (-2.83 to -2.07)
Zimbabwe	98.5(76.72 - 116.23)	2.64 (2.12 - 3.08)	179.81 (131.48-231.11)	2.92 (2.18-3.64)	0.74 (0.11 to 1.37)

ASR: Age-standardized rate; EAPC: Estimated annual percentage change.

e-Table 3 The DALYs and age-standardized DALYs rate of aneurysm in 1990 and 2017, and its temporal trends from 1990 to 2017 in 195 countries and territories.

Countries and territories	1990		2017		1990-2017
	DALYs	ASR per 100,000	DALYs	ASR per 100,000	EAPC
	No. $\times 10^3$ (95% UI)	No. (95% UI)	No. $\times 10^3$ (95% UI)	No. (95% UI)	No. (95% UI)
Afghanistan	1930.04(798.21 - 4216.74)	26.57 (11.59 - 57.69)	3482.75 (2160.9 - 6186.67)	26.92 (17.59-46.85)	0.08 (-0.03 to 0.19)
Albania	523.48(472.27 - 603.96)	21.82 (19.6 - 25.6)	846.5 (670.08 - 1046.66)	21.5 (17.01-26.54)	0.15 (-0.14 to 0.43)
Algeria	2584.52(1867.86 - 3672.01)	18.78 (13.5 - 26.6)	5872.84 (4282.52 - 8029.89)	17.27 (12.52-23.73)	-0.27 (-0.35 to -0.19)
American Samoa	17.33(14.33 - 19.63)	71.31 (59.61 - 81.13)	18.88 (16.28 - 23.23)	43.9 (38.19-53.23)	-2.12 (-2.46 to -1.79)
Andorra	69.7(50.21 - 90.54)	117.81 (85.59 - 152.52)	94.26 (67.21 - 123.38)	70.81 (50.37-93.06)	-2.17 (-2.32 to -2.02)
Angola	3842.72(2228.38 - 6214.07)	91.94 (57.62 - 143.46)	6840.07 (5197.07 - 9084.23)	61.62 (48.23-79.51)	-1.92 (-2.12 to -1.73)
Antigua and Barbuda	25.9(23.21 - 28.76)	48.61 (43.71 - 54.05)	34.11 (31.05 - 37.88)	34.54 (31.4-38.42)	-1.65 (-1.85 to -1.45)
Argentina	41134.29(37208.26 - 46278.35)	124.43 (112.73 - 139.75)	42613.85 (37740.93 - 48012.1)	80.31 (71.03-90.65)	-2 (-2.2 to -1.81)
Armenia	3437.97(3012.49 - 3986.99)	123.94 (108.62 - 143.83)	6249.36 (5832.53 - 6711.62)	150.34 (140.17-161.18)	0.79 (0.57 to 1.02)
Australia	24153.41(23191.45 - 25082.74)	117.33 (112.75 - 121.86)	17648.36 (15790.5 - 19749.12)	43.33 (38.68-48.55)	-4.33 (-4.56 to -4.1)
Austria	7892.72(7531.64 - 8262.68)	68.54 (65.46 - 71.58)	6882.64 (6339.58 - 7462.79)	42.03 (38.5-45.65)	-2.38 (-2.63 to -2.12)
Azerbaijan	1395.07(1152.15 - 1743.13)	25.87 (20.79 - 33.54)	3364.36 (2821.82 - 3874.4)	36.76 (30.37-42.41)	0.91 (0.68 to 1.15)
Bahrain	63.31(55.51 - 75.02)	28.45 (24.23 - 36.86)	178.45 (146.05 - 212.64)	16.94 (14.61-19.93)	-2.28 (-2.51 to -2.05)
Bangladesh	14691.52(9900.33 - 21403.43)	29.15 (20.04 - 42.62)	34488.43 (26812.66 - 44133.54)	28 (21.85-35.9)	0.04 (-0.14 to 0.21)
Barbados	153.44(135.27 - 173.04)	50.61 (44.9 - 56.58)	193.16 (171.49 - 217.42)	40.37 (35.86-45.35)	-1.24 (-1.41 to -1.06)
Belarus	6114.56(4904.83 - 7197.31)	46.59 (37.56 - 55.27)	8302.17 (7315.31 - 9519.17)	54.25 (47.5-62.86)	0.49 (0.18 to 0.81)
Belgium	12741.2(12063.26 - 13466.43)	80.94 (76.74 - 85.42)	9797.76 (8923.58 - 10737.07)	43.19 (39.28-47.39)	-2.82 (-3.02 to -2.61)
Belize	29.44(25.86 - 34.08)	30.32 (26.59 - 35.19)	79.66 (71.15 - 86.69)	30.61 (27.2-33.5)	-0.7 (-1.16 to -0.24)
Benin	1004.77(756.98 - 1335.98)	46.81 (35.05 - 61.94)	1586.96 (1102.5 - 2211.35)	32.2 (22.91-44.14)	-1.6 (-1.76 to -1.45)
Bermuda	89.39(72.05 - 104.47)	139.91 (112.98 - 163.36)	100.31 (90.93 - 110.2)	80.37 (72.85-88.21)	-2.28 (-2.4 to -2.15)
Bhutan	96.51(65.51 - 139.58)	36.55 (25.71 - 51.52)	269.83 (201.65 - 359.07)	43.93 (32.92-58.63)	0.78 (0.72 to 0.84)
Bolivia	1800.11(1009.9 - 2600.26)	50.61 (28.91 - 72.42)	3625.2 (2692.87 - 4683.2)	41.35 (30.83-53.48)	-0.73 (-0.77 to -0.69)
Bosnia and Herzegovina	2389.73(1958.81 - 2910.8)	54.52 (45.47 - 66.07)	3370.11 (2686.13 - 3816.21)	58.79 (47.52-66.44)	0.18 (-0.05 to 0.41)

Countries and territories	1990		2017		1990-2017
	DALYs	ASR per 100,000	DALYs	ASR per 100,000	EAPC
	No. $\times 10^3$ (95% UI)	No. (95% UI)	No. $\times 10^3$ (95% UI)	No. (95% UI)	No. (95% UI)
Botswana	379.96(293.52 - 476.94)	61.25 (48.06 - 75.73)	511.4 (403.69 - 666.17)	37.97 (30.18-49.25)	-1.92 (-2.04 to -1.81)
Brazil	88492.99(86384.89 - 90748.53)	88.53 (86.5 - 90.71)	210007.31 (200166.3 - 218697.83)	91.32 (87.08-95.03)	-0.18 (-0.34 to -0.01)
Brunei	128.83(97.28 - 160.85)	113.66 (87.22 - 141.78)	298.84 (265.27 - 337.99)	93.92 (83.74-106)	-0.59 (-0.78 to -0.41)
Bulgaria	4967.25(4584.22 - 5312.74)	42.1 (39.05 - 44.8)	6788.37 (6233.1 - 7381.31)	57.93 (53.08-63.11)	1.29 (1.01 to 1.56)
Burkina Faso	1787.55(1266.63 - 2419.3)	38.53 (27.29 - 51.75)	2793.22 (2045.2 - 3805.09)	31.02 (22.99-41.82)	-0.88 (-1 to -0.76)
Burundi	2500.29(1642.5 - 3639.23)	101.64 (70.25 - 146.11)	1720.99 (1184.07 - 2404.56)	38.82 (26.87-54.59)	-4.32 (-4.75 to -3.9)
Cambodia	1243.48(698.39 - 2049.37)	25.94 (15.41 - 42.25)	2800.89 (2273.91 - 3627.18)	25.27 (20.82-32.75)	-0.19 (-0.27 to -0.11)
Cameroon	2468.74(1890.89 - 3237.22)	52.18 (40.21 - 67.95)	4441.48 (3406.99 - 5771.88)	35.96 (27.7-46.55)	-1.64 (-1.78 to -1.49)
Canada	33437.42(32118.57 - 34803.55)	98.56 (94.82 - 102.54)	27289.68 (25023.05 - 29542.35)	41.59 (38.16-45.12)	-4.01 (-4.33 to -3.68)
Cape Verde	49.58(39.93 - 60.4)	21.03 (16.97 - 25.61)	109.11 (90.95 - 127.06)	24.36 (20.2-28.6)	0.46 (0.39 to 0.54)
Central African Republic	1311.92(666.75 - 2145.02)	105.97 (58.37 - 167.76)	1784.98 (950.06 - 2847.36)	77.26 (46.05-117.73)	-1.45 (-1.58 to -1.33)
Chad	1257.01(916.8 - 1636.46)	42.01 (30.65 - 54.64)	1869.19 (1372.85 - 2525.08)	32.84 (24.1-44.41)	-1.11 (-1.27 to -0.95)
Chile	5916.12(5530.49 - 6347.7)	56.05 (52.56 - 60.1)	12003.09 (10639.32 - 13509.02)	52.45 (46.53-59.17)	-0.13 (-0.34 to 0.09)
China	191163.4(157359.64 - 245087.71)	20.38 (16.61 - 25.58)	330759.23 (295635.53 - 367747.77)	17.24 (15.4-19.05)	-0.97 (-1.2 to -0.74)
Colombia	15181.96(14546.81 - 15885.24)	78.39 (75.06 - 82.42)	25809.99 (22387.47 - 29436.55)	48.15 (41.74-54.89)	-2.65 (-3 to -2.31)
Comoros	223.82(150.65 - 332.63)	99.39 (66.75 - 145.48)	204.62 (146.82 - 273.8)	43.91 (31.21-58.69)	-3.54 (-3.96 to -3.12)
Congo	1344(908.41 - 1988.27)	115.6 (81.53 - 168.55)	1871.41 (1410.52 - 2379.67)	73.53 (55.41-91.32)	-2.15 (-2.42 to -1.89)
Costa Rica	732.09(650.22 - 836.51)	38.14 (33.71 - 43.8)	2512.58 (2274.24 - 2779.83)	51.07 (46.22-56.46)	0.88 (0.67 to 1.09)
Cote d'Ivoire	2442.43(1753.36 - 3114.27)	52.22 (37.56 - 65.63)	4414.78 (3406.89 - 5597.99)	39.21 (30.53-49.41)	-1.4 (-1.59 to -1.22)
Croatia	2811.66(2653.44 - 2980.73)	43.43 (41.12 - 45.9)	5618.68 (5183.51 - 6086.37)	69.02 (63.56-75.15)	1.91 (1.53 to 2.29)
Cuba	9449.72(8559.47 - 10675.2)	88.79 (80.53 - 100.16)	11826.16 (10449.73 - 13502.99)	63.63 (56.26-72.62)	-1.51 (-1.65 to -1.36)
Cyprus	1039.09(822.03 - 1257.61)	120.54 (96.34 - 143.96)	1410.18 (1203.4 - 1664.43)	75.03 (64-87.54)	-2.12 (-2.5 to -1.75)
Czech Republic	6528.31(6149.59 - 6903.96)	48.15 (45.35 - 50.76)	10458.49 (9688.63 - 11347.16)	53.93 (49.92-58.4)	0.83 (0.61 to 1.05)

Countries and territories	1990		2017		1990-2017
	DALYs	ASR per 100,000	DALYs	ASR per 100,000	EAPC
	No. $\times 10^3$ (95% UI)	No. (95% UI)	No. $\times 10^3$ (95% UI)	No. (95% UI)	No. (95% UI)
Democratic Republic of the Congo	12124.69(8100.54 - 17548.07)	73.66 (49.05 - 104.35)	18389.86 (13361.36 - 24714.18)	51.85 (37.88-68.73)	-1.69 (-1.95 to -1.44)
Denmark	10379.37(9577.26 - 11158.29)	124.94 (115.4 - 134.12)	8642.64 (7860.44 - 9475.26)	75.21 (68.62-82.17)	-2.33 (-2.64 to -2.02)
Djibouti	147.41(91.28 - 221.14)	82.05 (51.38 - 121.25)	280.88 (178.48 - 398.53)	47.14 (30.32-66.74)	-2.79 (-3.14 to -2.43)
Dominica	59.43(52 - 66.89)	78.96 (69.63 - 88.75)	68.07 (61.47 - 74.77)	73.77 (66.66-81.24)	-0.48 (-0.61 to -0.36)
Dominican Republic	1288.69(1066.81 - 1471.92)	31.36 (26.14 - 35.76)	3649.86 (3014.15 - 4432.28)	39.09 (32.19-47.89)	0.81 (0.52 to 1.11)
Ecuador	2261.25(1996.41 - 2530.74)	37.73 (32.89 - 42.54)	5385.48 (4801.03 - 6076.43)	35.84 (32.02-40.35)	-0.15 (-0.35 to 0.05)
Egypt	7055.46(4391.28 - 12735.8)	23.01 (14.62 - 41.86)	18368.23 (10647.26 - 30167.67)	29.33 (17.27-47.71)	0.92 (0.83 to 1.01)
El Salvador	776.69(682.39 - 913.18)	23.62 (20.86 - 27.42)	1378.59 (1127.74 - 1665.16)	23.89 (19.55-28.77)	0.06 (-0.12 to 0.23)
Equatorial Guinea	225.62(118.02 - 384.2)	108.16 (61.11 - 178.74)	278.4 (186.95 - 396.63)	58.58 (40.09-82.05)	-2.85 (-3.22 to -2.48)
Eritrea	1333.04(731.47 - 2295.71)	117.02 (71.6 - 191.2)	1388.67 (873.46 - 1958.51)	52.28 (33.4-72.12)	-3.69 (-3.99 to -3.39)
Estonia	1278.89(1163.14 - 1403.76)	62.09 (56.59 - 68.15)	1400.57 (1180.15 - 1719.45)	58.02 (48.9-70.15)	-0.55 (-0.95 to -0.14)
Ethiopia	11465.1(5930.77 - 20474.76)	53.22 (30.96 - 90.26)	10227.36 (7062.62 - 13406.72)	24.55 (16.76-33.25)	-3.4 (-3.63 to -3.16)
Federated States of Micronesia	53.93(37.94 - 69.97)	102.98 (75.85 - 131.77)	58.02 (41.91 - 74.69)	79.25 (60.73-99.61)	-1.18 (-1.26 to -1.09)
Fiji	472.37(405.4 - 549.08)	116 (99.46 - 134.05)	812.32 (692.2 - 947.81)	108.6 (93.32-125.35)	-0.46 (-0.63 to -0.28)
Finland	11030.66(9974.49 - 12173.2)	152.27 (138.06 - 166.87)	8843.47 (8071.85 - 9753.22)	75.75 (69.28-83.27)	-2.59 (-2.74 to -2.45)
France	52034.82(49954.21 - 54315.62)	62.09 (59.6 - 64.69)	44524.47 (40505.48 - 48900.06)	34.39 (31.06-38.12)	-2.68 (-2.97 to -2.38)
Gabon	622.56(455.71 - 817.1)	105.59 (78.71 - 137.47)	762.59 (569.37 - 929.58)	71.75 (53.76-87.15)	-1.74 (-1.88 to -1.59)
Georgia	1312.76(1147.16 - 1502.33)	20.98 (18.51 - 23.89)	3648.54 (3327.17 - 4013.64)	66.63 (61.09-73.1)	6.16 (5.06 to 7.27)
Germany	86183.93(79850.84 - 92866.75)	67.88 (63.25 - 73.04)	75706.2 (67057.12 - 85258.46)	43.76 (38.77-49.34)	-1.76 (-1.9 to -1.61)
Ghana	3490.13(2814.86 - 4181)	49.49 (40.19 - 58.76)	5791.92 (4648.05 - 7007.08)	34.78 (28.29-42.29)	-2.14 (-2.78 to -1.49)
Greece	11373.18(10860.97 - 11943.92)	76.44 (73.14 - 80.32)	16887.15 (15586.36 - 18428.85)	81.28 (75.24-88.4)	-0.06 (-0.34 to 0.23)
Greenland	21.31(17.06 - 24.62)	61.84 (49.24 - 72.09)	23.64 (19.5 - 27.05)	36.49 (30.17-41.56)	-2.3 (-2.49 to -2.12)
Grenada	72.97(63.13 - 85.03)	102.11 (88.14 - 119.31)	127.65 (116.72 - 139)	84.85 (77.76-92.5)	-0.72 (-1 to -0.44)
Guam	107.03(76.48 - 130.55)	124.48 (90.24 - 149.48)	137.49 (112.77 - 197.45)	76.95 (63.49-110.02)	-2.14 (-2.34 to -1.94)

Countries and territories	1990		2017		1990-2017
	DALYs	ASR per 100,000	DALYs	ASR per 100,000	EAPC
	No. $\times 10^3$ (95% UI)	No. (95% UI)	No. $\times 10^3$ (95% UI)	No. (95% UI)	No. (95% UI)
Guatemala	1167.14(1026.47 - 1352.36)	30.04 (26.28 - 34.46)	2149.44 (1908.05 - 2438.09)	18.91 (16.77-21.36)	-2.4 (-2.72 to -2.08)
Guinea	1631.3(1247.84 - 2279.44)	46.54 (35.66 - 64.67)	2279.35 (1585.83 - 3187.51)	40.01 (27.9-55.23)	-0.6 (-0.85 to -0.34)
Guinea-Bissau	352.18(219.51 - 518.25)	79.62 (52.02 - 115.26)	385.38 (247.29 - 557.91)	48.82 (32.54-69.69)	-2.09 (-2.28 to -1.89)
Guyana	183.9(169.08 - 198.4)	44.8 (41.03 - 48.72)	378.39 (330.14 - 431.26)	61.54 (53.9-69.76)	1.21 (0.67 to 1.76)
Haiti	2507.1(1524.05 - 3576.87)	75.26 (47.81 - 106.75)	4495.79 (3462.97 - 5926.75)	67.26 (52.79-87.28)	-0.41 (-0.47 to -0.35)
Honduras	516.98(418.87 - 695.13)	22.33 (17.75 - 29.26)	1320.36 (1009.66 - 1786.96)	21.84 (16.64-29.35)	-0.21 (-0.36 to -0.05)
Hungary	8174.56(7843.8 - 8500.09)	56.08 (53.86 - 58.3)	9185.88 (8479.26 - 9899.2)	51.31 (47.34-55.34)	-0.34 (-0.57 to -0.12)
Iceland	217.89(201.63 - 234.16)	74.92 (69.28 - 80.36)	252.29 (232.15 - 274.05)	48.16 (44.22-52.27)	-2.06 (-2.27 to -1.84)
India	146872.32(106010.08 - 205338.01)	29.95 (22.24 - 41.64)	362069.72 (300333.76 - 435937.53)	33.13 (27.6-39.81)	0.24 (0.03 to 0.46)
Indonesia	25382.27(21186.74 - 31998.13)	24.88 (20.37 - 31.19)	60261.8 (49257.34 - 78069.81)	29.6 (24.14-38.35)	0.67 (0.6 to 0.74)
Iran	4940.03(4251.94 - 6041.05)	16.73 (14.28 - 20.4)	12840.46 (10741.89 - 13878)	17.81 (14.93-19.26)	0.57 (0.4 to 0.73)
Iraq	5546.76(4247.14 - 7000.63)	68.27 (53.55 - 84.79)	5917.65 (5264.66 - 6948.89)	25.02 (22.35-28.68)	-4.18 (-4.77 to -3.58)
Ireland	4225.19(4018.27 - 4450.68)	97.17 (92.47 - 102.25)	4393.46 (3947.85 - 4897.35)	60.41 (54.29-67.48)	-2.48 (-2.91 to -2.05)
Israel	1971.59(1821.57 - 2151.11)	39.41 (36.6 - 42.81)	2773.68 (2493.13 - 3070.57)	25.02 (22.46-27.68)	-2.17 (-2.37 to -1.98)
Italy	48885.56(46774.42 - 51086.88)	54.72 (52.42 - 57.03)	54222.94 (49586.61 - 59349.68)	40.37 (36.74-44.57)	-1.68 (-2.08 to -1.28)
Jamaica	668.53(596.81 - 762.13)	36.64 (32.73 - 41.72)	1370.64 (1118.8 - 1638.48)	47.63 (38.86-57.06)	0.56 (0.16 to 0.95)
Japan	98999.17(97047.52 - 101029.01)	57.94 (56.79 - 59.12)	229473.76 (216994.82 - 240683.29)	68.71 (64.74-72.68)	0.99 (0.81 to 1.18)
Jordan	763.67(516.61 - 985.25)	43.81 (29.87 - 59.05)	1885.33 (1574.92 - 2367.93)	29.93 (24.91-38.59)	-1.91 (-2.14 to -1.68)
Kazakhstan	5410.45(4731.71 - 6449.79)	38.4 (33.45 - 45.9)	8442.48 (7649.69 - 9563.82)	46.66 (42.55-52.29)	0.13 (-0.42 to 0.68)
Kenya	4485.9(3262.26 - 5564.98)	50.09 (36.06 - 63.78)	8567.69 (6731.09 - 10467.57)	38.2 (29.79-48.41)	-1.32 (-1.51 to -1.13)
Kiribati	15.41(12.9 - 17.51)	35.15 (29.83 - 39.59)	28.46 (22.35 - 34.71)	35.92 (28.79-43.54)	0.08 (0.04 to 0.12)
Kuwait	262.18(241.01 - 285.2)	27.53 (25.42 - 29.8)	742.14 (625.17 - 886.13)	25.32 (21.34-30.34)	0.08 (-0.35 to 0.51)
Kyrgyzstan	592.41(540.65 - 686.73)	18.48 (16.8 - 21.43)	766.28 (687.17 - 875.59)	16.21 (14.59-18.51)	-0.77 (-1.06 to -0.47)
Laos	1034.33(660.01 - 1661.02)	45.84 (31.17 - 72.52)	1535.14 (1158.13 - 2074.49)	35.46 (27.58-47.5)	-1.12 (-1.18 to -1.06)

Countries and territories	1990		2017		1990-2017
	DALYs	ASR per 100,000	DALYs	ASR per 100,000	EAPC
	No. $\times 10^3$ (95% UI)	No. (95% UI)	No. $\times 10^3$ (95% UI)	No. (95% UI)	No. (95% UI)
Latvia	1902.11(1727.9 - 2118.64)	52.52 (47.74 - 58.25)	1637.53 (1437.9 - 1853.97)	44.94 (39.17-51.18)	-0.96 (-1.34 to -0.58)
Lebanon	540.99(410.93 - 860.97)	22.82 (17.44 - 35.95)	1324.19 (999.44 - 1840.43)	21.04 (16.14-29.02)	-0.11 (-0.24 to 0.01)
Lesotho	509.34(383.52 - 659.95)	49.88 (37.82 - 64.38)	616.88 (426.42 - 804.2)	49.01 (34.59-63.05)	0.21 (0.01 to 0.4)
Liberia	621.8(413.63 - 821.22)	52.41 (35.02 - 69)	682 (484.2 - 917.32)	32.91 (23.72-43.8)	-1.97 (-2.3 to -1.64)
Libya	367.98(275.43 - 543.21)	16.7 (12.49 - 24.48)	1122.87 (820.1 - 1560.42)	21.74 (16.11-30.07)	1.19 (1.06 to 1.33)
Lithuania	2204.5(1982.69 - 2464.43)	48.28 (43.42 - 53.89)	2994.16 (2730.27 - 3257.71)	59.23 (53.77-64.78)	0.79 (0.38 to 1.2)
Luxembourg	434.45(404.15 - 465.33)	78.36 (72.93 - 84.08)	473.6 (411.12 - 543.65)	49.75 (43.17-57.16)	-2.37 (-2.68 to -2.07)
Macedonia	542.52(467.41 - 742.05)	28.37 (24.52 - 38.94)	1157.2 (877.42 - 1371.87)	35.56 (27-42.03)	0.95 (0.73 to 1.17)
Madagascar	7813.91(5074.17 - 11373.95)	126.55 (81.62 - 181.43)	9916.28 (6726.19 - 14132.41)	79.81 (54.25-111.3)	-2.24 (-2.47 to -2)
Malawi	2208.3(1102.86 - 3473.85)	50.79 (27.13 - 77.25)	3223.32 (2090.06 - 4411.49)	39.95 (26.07-54.41)	-1.55 (-1.96 to -1.14)
Malaysia	6974.04(5781.77 - 8025.93)	77.13 (62.31 - 88.95)	15910.44 (13369.94 - 18291.34)	64.85 (53.84-73.98)	-0.85 (-1.01 to -0.68)
Maldives	53.51(28.88 - 76.05)	61.01 (37.34 - 84.08)	94.05 (80.88 - 108.49)	33.54 (28.79-38.71)	-2.79 (-3.05 to -2.53)
Mali	2218.87(1346.72 - 3287.32)	52.5 (32.69 - 76.83)	2655.4 (1857.75 - 3718.1)	31.34 (21.89-43.35)	-2.29 (-2.57 to -2.01)
Malta	220.74(204.74 - 238.94)	50.64 (47.1 - 54.65)	336.17 (304.14 - 370.16)	38.96 (35.37-42.62)	-1.55 (-1.81 to -1.28)
Marshall Islands	21.84(12.9 - 29.89)	118.2 (72.63 - 160.29)	38.71 (23.94 - 52.99)	107.42 (71.2-143.67)	-0.52 (-0.69 to -0.36)
Mauritania	690.62(544.05 - 928.99)	63.82 (50.58 - 86.14)	683.67 (526.54 - 895.63)	33.82 (26.25-44.57)	-2.69 (-3 to -2.38)
Mauritius	271.95(251.52 - 294.05)	32.9 (30.43 - 35.59)	337.45 (305.82 - 372.17)	21.19 (19.25-23.23)	-1.77 (-1.96 to -1.57)
Mexico	11082.86(10793.02 - 11413.57)	23.39 (22.75 - 24.1)	19793.76 (18887.35 - 20654.36)	17.21 (16.41-17.93)	-1.32 (-1.45 to -1.18)
Moldova	1109.71(1001.62 - 1243.54)	24.19 (21.97 - 27.01)	1757.81 (1620.75 - 1914.98)	32.49 (29.98-35.38)	1.45 (1.06 to 1.85)
Mongolia	230.87(195.81 - 303.01)	20.5 (17.49 - 26.68)	466.57 (397.62 - 568.69)	19.11 (16.43-22.67)	-0.69 (-1.02 to -0.36)
Montenegro	960.27(848.31 - 1160.61)	147.93 (130.87 - 178.3)	1420.74 (1234.22 - 1633.18)	147.15 (127.91-168.85)	0.05 (-0.1 to 0.19)
Morocco	2977.92(2135.9 - 4487.03)	18.98 (13.42 - 28.43)	7163.85 (5257 - 10127.01)	22.09 (16.18-30.97)	0.53 (0.44 to 0.61)
Mozambique	5301.01(3234.72 - 7946.88)	79.31 (47.59 - 115.9)	8289.76 (5758.66 - 12109.35)	67.05 (47.25-95.51)	-0.63 (-0.77 to -0.49)
Myanmar	6751.01(4091.04 - 9956.51)	27.78 (17.75 - 40.72)	9984.41 (7673.09 - 12666.77)	22.74 (17.75-28.69)	-0.85 (-0.92 to -0.77)
Namibia	520.49(398.14 - 648.7)	69.29 (52.99 - 85.9)	597.91 (463.69 - 746.06)	42.11 (33.06-52)	-2.45 (-2.94 to -1.96)

Countries and territories	1990		2017		1990-2017
	DALYs	ASR per 100,000	DALYs	ASR per 100,000	EAPC
	No. $\times 10^3$ (95% UI)	No. (95% UI)	No. $\times 10^3$ (95% UI)	No. (95% UI)	No. (95% UI)
Nepal	2161.78(1499.42 - 3397.57)	21.91 (15.53 - 33.73)	5839.03 (4440.98 - 7598.18)	27.17 (20.9-35.17)	1.03 (0.78 to 1.27)
Netherlands	24100.35(22981.91 - 25210.34)	115.89 (110.63 - 121.36)	20258.9 (18622.04 - 21919.2)	59.82 (55-64.7)	-3.07 (-3.53 to -2.6)
New Zealand	6751.03(6427.61 - 7076.62)	163.53 (155.96 - 171.27)	5554.45 (5143.94 - 6008.76)	73.22 (67.83-79.11)	-3.63 (-3.86 to -3.39)
Nicaragua	339.24(292.09 - 384.33)	19.27 (16.39 - 21.88)	720.73 (619.31 - 865.34)	15.39 (13.25-18.29)	-1.03 (-1.15 to -0.92)
Niger	1332.48(960.08 - 1793.71)	43.13 (31.25 - 57.99)	1931.56 (1268.07 - 2764.13)	25.04 (17.02-35.26)	-2.38 (-2.65 to -2.11)
Nigeria	20861.07(14301.15 - 29616.61)	44.71 (30.97 - 62.71)	23143.98 (15422.16 - 37046.8)	27.3 (18.31-43.54)	-2.37 (-2.62 to -2.12)
North Korea	4687.55(3587.51 - 5965.39)	26.27 (20.65 - 32.88)	8507.09 (6954.13 - 10512.64)	26.99 (22.03-33.33)	-0.02 (-0.1 to 0.06)
Northern Mariana Islands	19.84(15.8 - 24.19)	93.66 (75.93 - 112.31)	22.24 (18.98 - 27.52)	43.26 (37.36-53.09)	-3.33 (-3.54 to -3.11)
Norway	8886.23(8645.38 - 9131.34)	126.22 (122.97 - 129.58)	7292.4 (6903.22 - 7724.22)	77.03 (72.75-81.72)	-2.3 (-2.6 to -1.99)
Oman	416.55(278.26 - 727.01)	47.89 (32.46 - 83.12)	960.4 (652.42 - 1441.35)	37.9 (26.13-55.23)	-0.95 (-1.14 to -0.75)
Pakistan	25869.53(20743.85 - 33779.09)	44.08 (34.87 - 57.65)	63470.06 (48614.67 - 80783.98)	56.7 (43.21-72.21)	0.73 (0.62 to 0.85)
Palestine	334.46(241.59 - 450.14)	33.9 (24.84 - 46.19)	706.81 (620.53 - 817.81)	27.71 (24.21-32.12)	-0.67 (-0.92 to -0.41)
Panama	815.81(734.72 - 897.82)	50.79 (45.62 - 56.06)	1454.44 (1311.88 - 1605.66)	36.65 (33.02-40.41)	-1.58 (-1.79 to -1.38)
Papua New Guinea	2405.69(1372.54 - 3589.94)	110.46 (68.91 - 161.32)	5058.58 (3146.21 - 7314.07)	95.45 (64.54-132.53)	-0.72 (-0.79 to -0.64)
Paraguay	1296.68(1083.2 - 1506.9)	54.77 (46.14 - 63.31)	3421.75 (2786.3 - 4225.6)	62.67 (51.23-77.32)	0.56 (0.35 to 0.76)
Peru	3656.53(3172.77 - 4338.1)	27.32 (23.65 - 32.6)	6230.68 (5076.92 - 7473.12)	20.11 (16.37-24.1)	-1.24 (-1.42 to -1.06)
Philippines	15196.75(13652.59 - 17261.47)	44.7 (39.54 - 51.51)	30614.72 (26390.98 - 35100.29)	40.99 (35.59-46.83)	-1.12 (-1.67 to -0.57)
Poland	32941.44(27833.53 - 38975.46)	72.69 (61.55 - 85.78)	40122.14 (37044.6 - 43413.39)	61.56 (56.73-66.63)	-0.61 (-0.88 to -0.34)
Portugal	5540.39(5294.43 - 5808.1)	40.95 (39.22 - 42.83)	6988.36 (6305.3 - 7679.12)	32.01 (28.71-35.36)	-1.22 (-1.42 to -1.03)
Puerto Rico	1364.24(1263.58 - 1474.22)	36.28 (33.63 - 39.16)	1300.49 (1179.07 - 1440.05)	19.45 (17.69-21.47)	-3.12 (-3.4 to -2.85)
Qatar	70.41(52.19 - 109.42)	47.98 (37.62 - 72.17)	242.42 (186.38 - 313.81)	20.8 (16.49-26.02)	-3.92 (-4.3 to -3.55)
Romania	11493.57(10300.16 - 12921.22)	41.38 (37.28 - 46.24)	15024.5 (14010.55 - 16100.3)	45.89 (42.76-49.16)	0.38 (0.21 to 0.54)
Russian Federation	112328.16(104476.84 - 119028.2)	61.01 (56.64 - 64.7)	172621.65 (166252 - 179497.47)	77.04 (74.06-80.42)	0.59 (0.28 to 0.91)
Rwanda	2471.26(1623.15 - 3816.76)	78.14 (51.35 - 116.87)	1722.51 (1186.32 - 2534.01)	29.52 (20.17-44.17)	-4.72 (-5.24 to -4.2)

Countries and territories	1990		2017		1990-2017
	DALYs	ASR per 100,000	DALYs	ASR per 100,000	EAPC
	No. $\times 10^3$ (95% UI)	No. (95% UI)	No. $\times 10^3$ (95% UI)	No. (95% UI)	No. (95% UI)
Saint Lucia	112.9(97.58 - 133.03)	121.93 (105.53 - 144.02)	187.54 (170.68 - 205.26)	90.83 (82.44-99.45)	-1.44 (-1.62 to -1.25)
Saint Vincent and the Grenadines	43.97(40.45 - 47.55)	57.26 (52.68 - 61.87)	72.34 (65.71 - 79.17)	53.48 (48.45-58.53)	-0.42 (-0.55 to -0.29)
Samoa	57.8(45.67 - 71.33)	66.32 (51.89 - 81.86)	72.76 (56.03 - 90.11)	53.95 (41.85-66.25)	-1.05 (-1.19 to -0.92)
Sao Tome and Principe	27.74(18.75 - 41.53)	39.3 (26.6 - 58.48)	38.19 (29.86 - 52.51)	34.89 (27.29-46.09)	-0.93 (-1.2 to -0.65)
Saudi Arabia	1135.95(817.13 - 1541.25)	16.99 (12.43 - 22.89)	4821.36 (3663.79 - 6325.47)	26.41 (20.55-32.53)	2.31 (2.03 to 2.59)
Senegal	1419.93(1057.84 - 1862.38)	40.98 (30.63 - 53.67)	1869.41 (1482.69 - 2355.89)	24.97 (19.85-31.37)	-2.33 (-2.68 to -1.99)
Serbia	7816.25(6496.56 - 9227.01)	66.77 (56.67 - 78.65)	11634.05 (8776.9 - 13415.72)	76.05 (57.3-87.65)	0.89 (0.74 to 1.04)
Seychelles	24.59(21.07 - 30.41)	41.99 (35.89 - 52.05)	37.68 (30.97 - 49.13)	35.92 (29.61-46.59)	-0.9 (-1.05 to -0.74)
Sierra Leone	1075.31(773.9 - 1410.03)	51.69 (36.97 - 67.55)	1414.39 (1103.5 - 1793.07)	37.96 (29.82-48.07)	-1.31 (-1.44 to -1.18)
Singapore	1551.05(1448.97 - 1658.03)	64.47 (60.42 - 68.39)	2120.44 (1917.77 - 2334.8)	31.19 (28.1-34.39)	-2.72 (-2.85 to -2.6)
Slovakia	2655.54(2155.83 - 4126.88)	44.57 (36.32 - 68.96)	3537.64 (2948.46 - 3959.68)	41.44 (34.27-46.24)	0.16 (-0.22 to 0.54)
Slovenia	1172.82(1091.4 - 1277.26)	47.27 (44.11 - 51.44)	1843.63 (1658.49 - 2042.29)	46.89 (42.15-51.99)	-0.35 (-0.87 to 0.17)
Solomon Islands	90.79(58.1 - 130.22)	59.92 (40.87 - 83.46)	168.43 (119.74 - 229.99)	49.07 (36.95-65.02)	-0.83 (-0.85 to -0.8)
Somalia	2682.49(1267.41 - 4854.93)	93.32 (50.18 - 155.97)	3446.48 (2218.07 - 5151.2)	49.16 (32.7-70.88)	-3.26 (-3.66 to -2.85)
South Africa	14310.93(11768.95 - 16114.44)	60.56 (48.5 - 70.39)	19696.04 (17899.78 - 23271.54)	43.65 (39.66-51.84)	-1.78 (-2.59 to -0.96)
South Korea	10361.88(8850.09 - 12036.19)	33.61 (29.23 - 38.95)	19533.09 (17462.47 - 21794.36)	23.72 (21.28-26.45)	-1.37 (-1.49 to -1.25)
South Sudan	2091.87(1054.68 - 3691.73)	81.34 (44.65 - 137.1)	1691.48 (1033.7 - 2616.47)	42.34 (26.97-63.94)	-3.2 (-3.57 to -2.82)
Spain	26816.07(25636.21 - 28075.11)	48.77 (46.74 - 50.98)	35666.26 (32325.85 - 39247.35)	39.63 (35.81-43.61)	-1.42 (-1.88 to -0.96)
Sri Lanka	3692.11(2969.23 - 4540.92)	33.29 (27.6 - 40.28)	6783.51 (5197.23 - 8531.62)	28.58 (22.07-35.9)	-0.77 (-1.07 to -0.47)
Sudan	2182.1(1427.05 - 3719.32)	20.86 (13.64 - 34.96)	4426.22 (2984.54 - 6749.59)	22.12 (14.75-33.73)	0.26 (0.14 to 0.39)
Suriname	133.18(117.13 - 150.01)	50.56 (44.27 - 57.18)	264.81 (230.8 - 301.63)	45.98 (40.12-52.26)	-0.73 (-0.95 to -0.52)
Swaziland	196.67(155.74 - 238.12)	62.05 (49.4 - 74.93)	314.76 (232.23 - 400.55)	51.49 (38.78-64.35)	-0.36 (-0.72 to 0)
Sweden	18396.59(17704.88 - 19132.38)	120.61 (116.31 - 125.36)	14531.07 (13541.54 - 15645.57)	71.15 (66.35-76.52)	-2.41 (-2.78 to -2.04)
Switzerland	8478.68(7474.48 - 9474.92)	80.04 (70.61 - 89.33)	7214.13 (6567.77 - 7905.84)	42.51 (38.51-46.69)	-2.59 (-2.72 to -2.46)

Countries and territories	1990		2017		1990-2017
	DALYs	ASR per 100,000	DALYs	ASR per 100,000	EAPC
	No. $\times 10^3$ (95% UI)	No. (95% UI)	No. $\times 10^3$ (95% UI)	No. (95% UI)	No. (95% UI)
Syria	1943.23(1348.85 - 2748.68)	33.35 (23.9 - 47.84)	4029.36 (3268.02 - 5043.5)	31.01 (25.29-38.42)	-0.8 (-1.05 to -0.55)
Taiwan (Province of China)	4446.26(4228.47 - 4669.36)	26.12 (24.83 - 27.48)	19213.94 (17721.83 - 20796.11)	52.23 (48.22-56.46)	2.66 (2.28 to 3.05)
Tajikistan	873.22(751.46 - 1026.58)	30.33 (25.9 - 35.83)	1863.22 (1633.41 - 2089.86)	35.5 (30.06-39.58)	0.01 (-0.3 to 0.33)
Tanzania	6931.21(4429.21 - 9646.93)	60.6 (39.25 - 82.58)	11500.04 (7995.22 - 15929.09)	46.74 (32.71-65.87)	-1.65 (-1.98 to -1.32)
Thailand	12054.32(9419.3 - 15347.19)	34.15 (26.84 - 41.72)	26976.39 (23062.34 - 30275.35)	28.83 (24.78-32.33)	-1.24 (-1.45 to -1.03)
The Bahamas	127.86(114.39 - 144.72)	78.07 (70.1 - 88.28)	251.03 (226.75 - 279.08)	67.3 (60.74-74.88)	-0.59 (-0.68 to -0.51)
The Gambia	218.03(152.19 - 298.15)	55.97 (39.53 - 74.73)	397.71 (302.55 - 519.77)	40.04 (30.8-51.12)	-1.39 (-1.52 to -1.25)
Timor-Leste	74.06(52.79 - 105.69)	23.04 (16.69 - 32.17)	205.14 (144.97 - 327.18)	25.69 (18.6-40.5)	0.38 (0.24 to 0.52)
Togo	733.15(557.16 - 956.59)	53.4 (40.44 - 69.12)	1396.59 (1054.38 - 1809.67)	37.66 (29.17-47.99)	-1.54 (-1.69 to -1.38)
Tonga	22.76(18.95 - 27.18)	39.69 (33.54 - 47.02)	32.73 (26.15 - 40.12)	40.12 (32.13-49.05)	0.11 (0.05 to 0.16)
Trinidad and Tobago	912.16(831.46 - 1014.87)	103.02 (94.04 - 114.44)	1449.74 (1203.63 - 1753.69)	80.83 (67.17-97.33)	-1.3 (-1.54 to -1.07)
Tunisia	881.46(661.62 - 1300.75)	16.91 (12.75 - 24.98)	2146.43 (1483.59 - 3050.93)	17.81 (12.36-25.25)	-0.09 (-0.25 to 0.07)
Turkey	23173.16(16283.89 - 31020.7)	60.4 (42.71 - 82.14)	45210.86 (39340.28 - 53041.41)	51.49 (44.87-60.7)	-0.55 (-0.91 to -0.2)
Turkmenistan	500.51(454.92 - 566.89)	23.98 (21.69 - 27.57)	2017.76 (1706.62 - 2409.78)	49.54 (41.37-60.07)	2.85 (2.41 to 3.29)
Uganda	3415.02(2255.38 - 4900.52)	48.9 (31.63 - 70.43)	4745.01 (3177.31 - 6919.86)	32.65 (21.58-47.31)	-2.27 (-2.66 to -1.87)
Ukraine	36199.84(32968.03 - 40850.98)	49.67 (45.49 - 55.72)	45982.71 (42621.23 - 49380.6)	65.23 (60.27-70.1)	0.5 (0.09 to 0.92)
United Arab Emirates	212.23(137.94 - 361.12)	30.95 (18.53 - 56.41)	2405.6 (1640.4 - 4061.92)	43.77 (28.54-77.9)	1.32 (1.15 to 1.48)
United Kingdom	147048.57(144766.27 - 149332.83)	151.65 (149.41 - 153.94)	100065.86 (97627.66 - 102955.77)	78.15 (76.27-80.36)	-3.2 (-3.58 to -2.83)
United States	287172.08(282468.31 - 292258.78)	87.32 (85.86 - 88.89)	222449.6 (214470.2 - 230605.76)	44.21 (42.52-45.9)	-3.23 (-3.5 to -2.95)
Uruguay	4700.7(4310.57 - 5101.44)	120.26 (110.36 - 130.21)	4906.37 (4336.69 - 5514.97)	97.08 (85.78-109.45)	-1.02 (-1.17 to -0.88)
Uzbekistan	1337.14(1060.1 - 1611.14)	11.05 (8.57 - 13.54)	4958.23 (4350.56 - 5639.7)	22.8 (20.14-25.96)	3.18 (2.97 to 3.38)
Vanuatu	71.94(50.88 - 98.93)	94.9 (67.97 - 127.6)	152.94 (104.83 - 232.19)	85.32 (59.16-128.3)	-0.5 (-0.55 to -0.45)
Venezuela	5287.37(4741.47 - 5847.16)	51.38 (46.12 - 56.93)	11603.18 (9852.66 - 13527.05)	41.58 (35.43-48.55)	-1.28 (-1.49 to -1.07)

Countries and territories	1990		2017		1990-2017
	DALYs	ASR per 100,000	DALYs	ASR per 100,000	EAPC
	No. $\times 10^3$ (95% UI)	No. (95% UI)	No. $\times 10^3$ (95% UI)	No. (95% UI)	No. (95% UI)
Vietnam	10925.14(8690.25 - 14948.1)	26.11 (20.57 - 35.47)	22940.78 (18831.3 - 28971.69)	25.77 (21.27-32.42)	-0.21 (-0.29 to -0.13)
Virgin Islands, U.S.	54.02(48.22 - 60.49)	63.09 (56.09 - 70.45)	109.08 (87.96 - 126.23)	57.26 (46.95-65.64)	-0.54 (-0.71 to -0.38)
Yemen	1389.09(687.27 - 2847.34)	23.71 (12.96 - 47.51)	3873.7 (2665.87 - 5850.44)	26.86 (18.5-40.05)	0.38 (0.29 to 0.47)
Zambia	1947.86(1318.46 - 2826.19)	62.41 (41.85 - 86.92)	2849.41 (2030.84 - 3741.04)	41.2 (29.13-53.08)	-2.5 (-2.93 to -2.08)
Zimbabwe	2451.8(1875.93 - 2900.95)	53.48 (41.08 - 63.12)	5012.9 (3581.65 - 6633.52)	63.87 (46.59-82.53)	1.14 (0.37 to 1.91)

ASR: Age-standardized rate; DALY: Disability-adjusted life year; EAPC: Estimated annual percentage change.
